# Traditional Meiyu–Baiu has been suspended by global warming

**DOI:** 10.1093/nsr/nwae166

**Published:** 2024-05-15

**Authors:** Zhicong Yin, Xiaolei Song, Botao Zhou, Wenhao Jiang, Huopo Chen, Huijun Wang

**Affiliations:** Key Laboratory of Meteorological Disaster, Ministry of Education / Collaborative Innovation Center on Forecast and Evaluation of Meteorological Disasters, Nanjing University of Information Science & Technology, Nanjing 210044, China; Nansen-Zhu International Research Centre, Institute of Atmospheric Physics, Chinese Academy of Sciences, Beijing 100029, China; Key Laboratory of Meteorological Disaster, Ministry of Education / Collaborative Innovation Center on Forecast and Evaluation of Meteorological Disasters, Nanjing University of Information Science & Technology, Nanjing 210044, China; Key Laboratory of Meteorological Disaster, Ministry of Education / Collaborative Innovation Center on Forecast and Evaluation of Meteorological Disasters, Nanjing University of Information Science & Technology, Nanjing 210044, China; Key Laboratory of Meteorological Disaster, Ministry of Education / Collaborative Innovation Center on Forecast and Evaluation of Meteorological Disasters, Nanjing University of Information Science & Technology, Nanjing 210044, China; Nansen-Zhu International Research Centre, Institute of Atmospheric Physics, Chinese Academy of Sciences, Beijing 100029, China; Key Laboratory of Meteorological Disaster, Ministry of Education / Collaborative Innovation Center on Forecast and Evaluation of Meteorological Disasters, Nanjing University of Information Science & Technology, Nanjing 210044, China; Nansen-Zhu International Research Centre, Institute of Atmospheric Physics, Chinese Academy of Sciences, Beijing 100029, China

**Keywords:** global warming, Meiyu–Baiu, extremes, human activity, culture and civilization

## Abstract

More than 1000 years, the Meiyu–Baiu have shaped the uniqueness of natural resources, civilization and culture in the Yangtze River Basin of China and the main islands of Japan. In recent decades, frequent rainstorms and droughts have seemingly diminished the misty features of traditional Meiyu–Baiu rainfall. However, there is still no consensus on whether their traditional nature is suspended. In this study, we quantitatively demonstrate that the Meiyu–Baiu almost completely lost their traditional features during 1961–2023, ∼80% of which can be attributed to anthropogenic warming. Furthermore, in a warmer future, the traditional Meiyu–Baiu will be more unlikely to appear. This study underscores the urgency in adapting to climate shift because destructive extremes are measurably taking the place of mild and maternal rains.

## Teaser

The oriental Meiyu-Baiu has almost completely lost and will keep away from its original and intrinsic features known as “misty rains”, which are attributed to anthropogenic activities

## INTRODUCTION

China's Yangtze River Basin and Japan are globally important commercial and cultural centers, and home to ∼500 million people (Fig. S1). In these regions, a distinct and far-reaching meteorological phenomenon occurs during early summer that has attracted worldwide attention. In China, this phenomenon is called the Meiyu, while in Japan it is referred to as the Baiu [1–3]. The traditional characteristics of the Meiyu–Baiu have long been identified as weather with gentle and successive rain, muggy and high-humidity environments, and short sunshine duration [4,5]. Indeed, descriptions of the Meiyu in Chinese literature date back to at least the Jin Dynasty (265–420 AD) and, since the Tang Dynasty (618–907 AD), numerous oriental poems, paintings and historical documents have consistently expressed feelings towards ‘misty rains’ (Table S1). For example, Du Fu, a very famous poet, created a poem named *Meiyu* in ∼760 AD and spoke of ‘a fine rain like smoke and mist comes and continues for several days’. Therefore, the traditional Meiyu–Baiu with significantly misty features has had a long course in Asian history.

**Figure 1.**
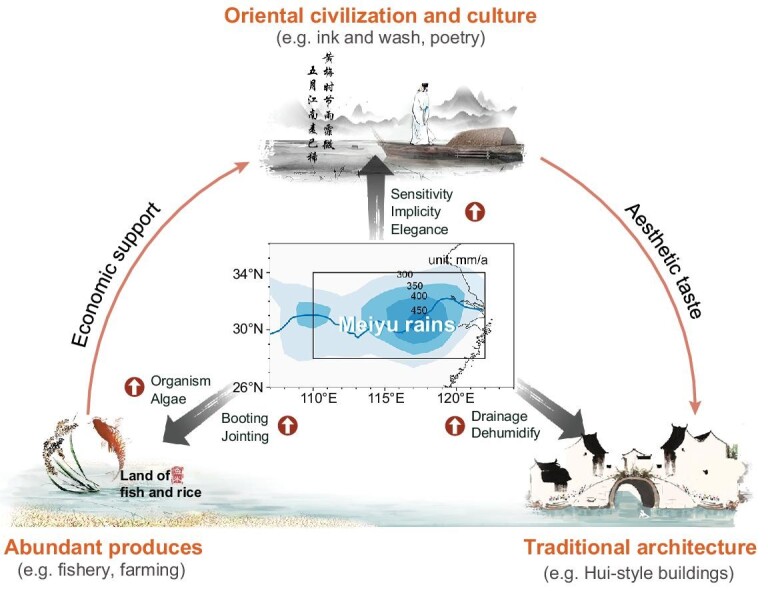
Conceptual framework of how the traditional Meiyu has shaped the unique Orient for >1000 years. The most important socio-economic aspects in the Yangtze River basin are agro-ecosystem, culture and architecture. The arrows indicate the connections between the different aspects and the rounded arrows indicate increases in parameters.

Climate conditions have been widely recognized as a crucial factor shaping human society and culture [6,7]. In this respect, the conceptual framework in Fig. 1 shows how the traditional Meiyu rain has profoundly shaped the unique Orient for >1000 years. Although the traditional Meiyu are closely linked to moldy damage, they do not frequently cause extreme flooding events. During the Meiyu period, local paddy rice is in its jointing and booting stages when its water demand is particularly high [8]. Therefore, the continuous and gentle rain at this time of year is highly conducive to strong levels of rice production. Meanwhile, the warm and moist conditions also promote the growth of organisms and algae in lakes and rivers [9,10], which provide an abundance of food to fisheries. Hence, the Meiyu region is often called ‘the land of fish and rice’ (Fig. 1). These material abundances have also supported splendid culture developments. In addition, rain like thin smoke and fog has shaped the behavioral traits of local inhabitants to be more sensitive, implicit and elegant [11,12]. Also, the physical beauty of the traditional Meiyu became a frequently used literary image. Thus, its misty nature has inspired distinctively euphemistic poetry and ink-and-wash paintings in this area (Table S1 and Fig. S2). These cultural aspects are entwined with improvements in the management of agriculture and fisheries, as well as the construction of characteristic Hui-style architecture and Suzhou-style gardens (Fig. 1). Taking Hui-style buildings as an example, the walls are coated with white lime and the rooftops are up-warped to enhance their rainproofing and ability to dry out (Fig. 1). Similarly, in Japan, the exploitation and management of natural resources and the development of civilization and culture are also influenced by the traditional Baiu rain [13].

The Meiyu and Baiu have undergone major changes under global warming. During the mid-twentieth century, the traditional Meiyu–Baiu was predominant [14]. For example, in 1962, light rain lasted for more than a week in Nanjing (32.05°N, 118.45°E) and the apparent temperature (AT) was ∼29.5°C, with the relative humidity no drier than 75% (Fig. S3a). Similar, non-disastrous conditions were also observed in 1972 and 1987 (Fig. S3b and c). However, in more recent decades, both the Meiyu and the Baiu have seldom exhibited their traditional misty features. In 2020, a super-strong Meiyu and Baiu triggered devastating floods in eastern China and Japan (Fig. S4a) [15]. Conversely, in the summer of 2022, a long-lasting and blistering heatwave and drought covered the entire Yangtze River Basin and Japan islands (Fig. S4b) [16]. Then, in the summer of 2023, heavy rain and sunny hot weather appeared alternately, which also did not conform to the traditional characteristics of the Meiyu (Fig. S4c). In contrast to the traditional Meiyu–Baiu, the extreme drought and rainfall pose considerable threats to biodiversity and grain production [17], and also have significant impacts on humans’ mind and body [18]. The above-mentioned meteorological conditions are unfavorable for the generation of mold—a notable indicator of the traditional Meiyu–Baiu (Table S2) [19]. However, owing to the lack of quantitative metrics applicable to misty rains, there is still no consensus on whether the traditional Meiyu–Baiu has been declining significantly.

The Meiyu–Baiu is a complex weather system and climate phenomenon that cannot be identified and portrayed by a single variable [20,21]. As simple examples, two kinds of untraditional Meiyu [i.e. heavily rainy (2020) and hot–dry (2022)] are always located on the two opposites of each curve in Fig. S5, which significantly influences the trend detection and needs to be reconciled. The rich dimensions of the Meiyu–Baiu include its rainfall, humidity, air temperature and solar radiation (Table S2). Many previous studies have pointed out how one of these features changed in the past and or is expected to change in the future [22]. However, these studies have tended to focus on increases in extreme events (rainstorms or heatwaves) and are unable to directly transfer to the changes in traditional misty rain and associated connotations. There is currently no robust understanding of whether and to what extent the changes in the traditional Meiyu–Baiu can be attributed to climate warming.

Accordingly, for the first time, we constructed a 3D index to quantify the deviation degree of misty rain (D2MR) in each year and investigated the changes using site observations in the Meiyu–Baiu period (from 10 June to 17 July) during 1961–2023. In this study, the Meiyu and Baiu region were defined as the Yangtze River Basin in China (28°–34°N, 110°–122.5°E) and the main islands of Japan (28°–44°N, 129°–146°E). Furthermore, we performed detection and attribution analyses to explore the human influences in the historical period on the basis of Coupled Model Intercomparison Project Phase 6 (CMIP6) simulations [23,24]. We also projected how the traditional Meiyu–Baiu are expected to vary in the future under different Shared Socioeconomic Pathway (SSP) scenarios [25].

## SUBSTANTIALLY WEAKENED MISTY FEATURES OF THE MEIYU–BAIU

The sub-indexes to describe each dimension of the Meiyu–Baiu were redefined and are different from previous studies: (i) Consecutive days without rain (CDnoR): the sum of rainless days that lasted for ≥3 days, aimed at identifying effective breaks in rainfall (Fig. S6a). A transient period of 1–2 clear or cloudy days does not change the continuity of rainfall in the Meiyu–Baiu [26]. Although the mean state in 2023 seemingly was not far from being one of misty rain (Fig. S5), the frequent disappearance of rainfall for 3 days efficiently interrupted the processes of moldy generation and could not be classed as a traditional Meiyu–Baiu (Fig. S4c). (ii) Rainfall intensity in 80% rain days (RI80R): the daily rainfall intensity after removing the top 20% of rainy days (Fig. S6b). The reason why we only retained 80% of rainy days was to exclude conditions that rainstorms of 2–3 days alter the basic features of the Meiyu–Baiu. If we did not set this threshold, then the year 2022, with 2 heavy-rain days, would possibly have been misjudged as light rain even though hot–dry disasters were occurring (Fig. S4b). On the other hand, the misty features might also have been falsely weakened by several heavy-rain days (e.g. 3 years in Fig. S3). (iii) Hot days with AT larger than its 70% quantile (HD70AT): the sum of hot days with AT larger than its 70% quantile value (Fig. S6c). When the AT was greater than this threshold, nearly 60% of the days showed no rain, while, when the AT was less, only 40% showed no rain, and the air temperature difference between the two categories was ∼5°C (Fig. S7). The years with greater HD70AT included hot–dry years (e.g. 2022) and effectively excluded the years cooled by heavy rain (e.g. 2016, 2020).

Although the above three sub-indexes are appropriately and physically designed, each one alone could not fully separate the years of the traditional Meiyu–Baiu, severe drought and heavy rain. Therefore, the Meiyu–Baiu in each year was assigned a 3D coordinate (CDnoR, RI80R, HD70AT) and the D2MR index was calculated as the Euclidean distance [27] to a defined standard misty rain (SMR) and can be calculated using the following equation:


\begin{eqnarray*}
{\mathrm{D2MR}} = \sqrt {{{{\mathrm{({CDnoR}\\!-\\!CDno}}{{\mathrm{R}}_{{\mathrm{SMR}}}}{\mathrm{)}}}^{\mathrm{2}}}{\mathrm{ + (RI80R\\!-\\!RI80}}{{\mathrm{R}}_{{\mathrm{SMR}}}}{{\mathrm{)}}^{\mathrm{2}}}{\mathrm{ + (HD70AT\\!-\\!HD70A}}{{\mathrm{T}}_{{\mathrm{SMR}}}}{{\mathrm{)}}^{\mathrm{2}}}}
\end{eqnarray*}

where SMR are defined as the average of the first decade. Before calculating the D2MR index, the three sub-indexes were standardized to make them comparable.

**Figure 2.**
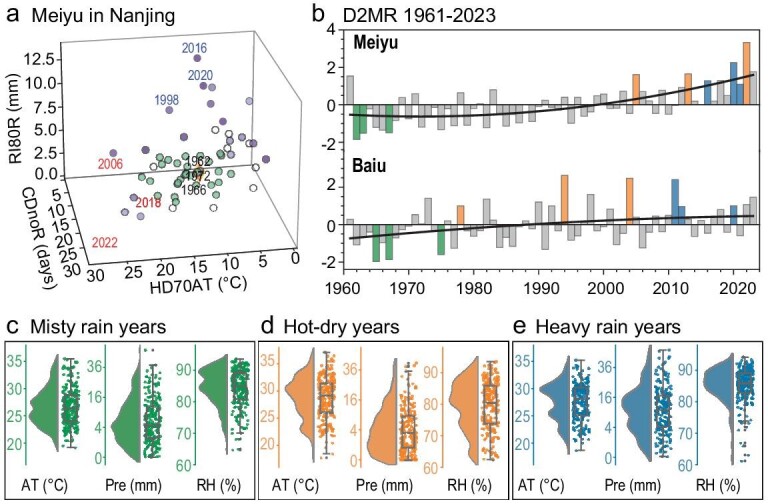
Changes in Meiyu–Baiu features. (a) 3D plots of three sub-indexes of D2MR (dots) in Nanjing during 1961–2023. The blue and green dots indicate years in which D2MR was greater or less than ±0.2 standard deviations. The yellow rhombus indicates SMR. Red, blue and black years represent hot–dry, heavy-rain and traditional Meiyu years, respectively. (b) Variation in D2MR (bars) averaged in the Meiyu and Baiu regions from 1961 to 2023 and their quadratic trends (black curves). The density distribution of weather conditions (apparent temperature (AT), precipitation (Pre) and relative humidity (RH)) in three representative years of (c) traditional Meiyu**–**Baiu rain (green bars in (b)), (d) hot–dry conditions (orange bars in (b)) and (e) heavy rain (blue bars in (b)).

Figure 2 shows the 3D coordinates of the Meiyu in Nanjing and the average of 1961–70 was selected as the SMR (yellow rhombus in Fig. 2a and Movie S1). During the years with smaller D2MR (green dots in Fig. 2a and Movie S1), it is evident that Nanjing experienced light rain on most days (Fig. S8a) and was muggy with a hot–wet overlying atmosphere (Fig. S8b and c), indicating the misty characteristics of the traditional Meiyu. In contrast, the untraditional Meiyu, which is further away from the SMR, can be divided into two categories: extreme heavy rain and hot–dry events (blue dots in Fig. 2a and Movie S1). To assess whether the results are sensitive to the thresholds of the sub-indexes and selection of SMR and sites, we perturbed them and found that the annual distributions and long-term trends of D2MR were consistent (Figs S9 and S10).

**Figure 3.**
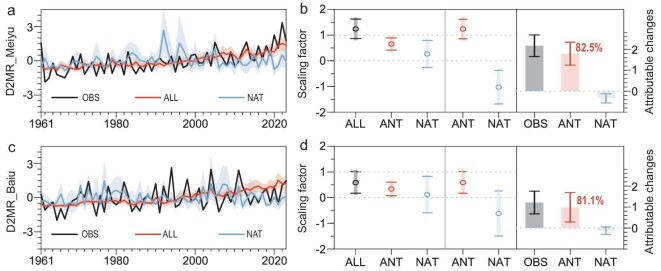
Attribution of the D2MR changes over the Meiyu and Baiu regions. D2MR in observation (OBS) and MME under the ALL and NAT forcings in the (a) Meiyu and (c) Baiu regions during 1961–2023. Shadows represent the 5%–95% ranges of ensemble simulations. (b and d) The best estimates of the scaling factors *β* (data points) and corresponding 5%–95% uncertainty ranges (error bars) derived from the single-signal detection analysis (left axis) and the two-signal (ANT and NAT) analysis (middle axis). The attributable changes (right axis) originated from the two-signal analysis.

During early summer, D2MR showed significant variations in the Meiyu–Baiu region (Fig. 2b). On the regional scale, two kinds of extremely untraditional Meiyu–Baiu were uniformly defined by larger D2MR, i.e. 3 hot–dry years (orange bars in Fig. 2b) and 3 heavy-rain years (blue bars in Fig. 2b). Three years with the smallest D2MR were the most representative of traditional Meiyu–Baiu years (green bars in Fig. 2b) and the associated meteorological conditions were well in accordance with the definition of the traditional Meiyu–Baiu (i.e. muggy but not too hot and persistent gentle rain or cloudy; Fig. 2c). Even when the daily observations at all sites were plotted, significant differences could be recognized from the classifications based on D2MR (Fig. 2c–e). The median AT in hot–dry years was 2.8°C higher than that of traditional Meiyu–Baiu years, but the relative humidity decreased by 4.5% and precipitation decreased by ∼18%. The heavy-rain years had 45% more precipitation than traditional Meiyu–Baiu years and most of the relative humidity was concentrated at a higher level.

For both the Meiyu and the Baiu, the area-mean D2MR presented a significant upward trend during 1961–2023 (*P* < 0.01), which showed that the misty features had reliably receded. Such an increasing trend of D2MR was also observed at most of the sites. The most representative traditional Meiyu–Baiu was mainly before the mid-1970s. Most extreme hot–dry years and heavy-rain years were concentrated after 2010 in the traditional Meiyu region, while those in the traditional Baiu region spread from 1990 to 2023. The linear trend of the normalized Meiyu D2MR (0.34/10a) was much stronger than that of the Baiu (0.19/10a). This difference in changing trend not only meant that the weakening of misty features was more significant in the Meiyu region than in the Baiu region, but also indicated that climate extremes were much more severe in the Meiyu region. The uptrend was further enhanced during 2010–23 (Fig. 2b) and indicated that the misty features had almost completely disappeared, especially in the Meiyu region.

The trends of normalized CDnoR, RI80R and HD70AT were all upward (*P* < 0.05) except the Baiu CDnoR (Fig. S11). In the Meiyu region, RI80R accounted for most of the D2MR changes, indicating that the strengthened rainfall intensity was the main reason for the decrement of misty features. The increasing discontinuity of rainfall and AT also obviously contributed to the upward D2MR trend (Fig. S11a and e). Of particular note is that CDnoR seemed to have two phases: lightly negative before 2000 and largely positive after that (Fig. S11a). In the Baiu region, the enhancement of rainfall intensity and AT equally elevated D2MR (Fig. S11d and f); however, CDnoR showed interdecadal variations and added little to the rising D2MR in the near decade (Fig. S11b). The Meiyu–Baiu period varies slightly from year to year [26]. To assess whether D2MR and the three sub-indexes were sensitive to the selection of the Meiyu–Baiu period, we extended it to the whole of June and July, and found that the changing trend remained significant (above the 95% confidence level), for both the Meiyu and the Baiu (Fig. S12). Therefore, both of the traditional Meiyu and Baiu had faded away and we further investigated the reasons behind these significant trends.

## ATTRIBUTION STUDIES

State-of-the-art CMIP6 simulations were employed to detect and attribute the observed changes of D2MR, for which the total number of models was 24 (Table S3). CMIP6/ALL encompasses a comprehensive assessment that accounts for the anthropogenic climate forcings (emissions of greenhouse gases and aerosols), as well as the natural climate forcings (NAT: solar and volcanic activities). In CMIP6/ALL simulations, each model (Fig. S13) and its multi-model ensemble (MME) were able to elegantly capture the upward trajectories of both the Meiyu and the Baiu D2MR (Fig. 3a and c). Furthermore, the MME trend of D2MR was stronger in the Meiyu region than in the Baiu region, which was also consistent with observations (Fig. S13). In contrast, the increasing D2MR trends of the Meiyu and Baiu were not successfully reproduced by the CMIP6/NAT ensemble simulations (Fig. 3a and c). The best estimates of scaling factors, implemented by the optimal fingerprinting method (Supplementary Methods), showed that only the anthropogenic (ANT) signal (ALL-NAT; anthropogenic forcings) was detectable, accounting for 82.5% (75.2%–87.2%) and 81.1% (42.6%–96.1%) of the observed Meiyu and Baiu trends, respectively (Fig. 3b and d). Combining the D2MR of Meiyu and Baiu, the detection and attribution results remained similar, and the contribution of the ANT signal was particularly pronounced, soaring to 90.7% (76.8%–99.8%). Therefore, the suspension of traditional Meiyu–Baiu in recent decades was significantly influenced by anthropogenic climate change.

During the Meiyu–Baiu period, CMIP6/ALL also captured the observed increasing trend of HD70AT well, in which 150.8% and 113.2% of anthropogenic contributions could be detected in the Meiyu and Baiu regions, respectively (Fig. S14a and b, and Fig. S15a and b). The simulated RI80R was also consistent with the observations, but the changing trend was much smaller than that of HD70AT. The ANT signal could be detected in the Meiyu RI80R with a contribution of 107.2%, while the ANT signal could be detected but with much lower contributions in the Baiu (Fig. S14e and f, and Fig. S15e and f). However, the increasing trend of CDnoR was captured in the NAT forcing simulation, but only in the Meiyu region (Fig. S14c), which was estimated to contribute by 40.7% (Fig. S14d). In both the ALL and NAT simulations, the Baiu CDnoR did not show an upward trend, which may be related to the weak trend but strong interdecadal variations in observed CDnoR (Fig. S15c). Consequently, no external forcing signal could be detected (Fig. S15d), implying that the Baiu CDnoR may be mainly affected by internal variability.

In general, the anthropogenic contributions were similar to the observed changes in D2MR in the Meiyu (82.5%) and Baiu (81.1%) regions, but the respective attribution results for the sub-indexes showed obvious diversities. Anthropogenic climate change significantly amplified the strong anomalies of HD70AT and RI80R, and natural forcing also made some contributions to the CDnoR changes in the Meiyu region. In contrast, the contributions of anthropogenic signals in the Baiu D2MR were relatively modest and possessed greater uncertainties. This difference likely arose from the limited impact of human activities on the RI80R changes and the undetectable CDnoR (Fig. S15d and f). Notably, previous research has shown that ANT signals can be detected in the total summer precipitation and the intensity of heavy precipitation in East Asia, and no significant ANT signal is observed in moderate and light precipitation [28]. However, the ANT influences on the traditional features of the Meiyu and Baiu can be significantly detected and attributed in this study, indicating the need to examine each dimension of the Meiyu–Baiu and the advantages of constructing a deviation degree index.

## Shut-OFF OF TRADITIONAL MEIYU–BAIU IN THE FUTURE

The aforementioned detection findings imply that the ALL forcing simulations tend to overestimate the D2MR changes of the Baiu in future projections but underestimate those of the Meiyu (Fig. S16). Therefore, we applied an observation-constrained framework based on the best scaling factors to project the changes in D2MR until the year 2100 (Supplementary Methods).

**Figure 4.**
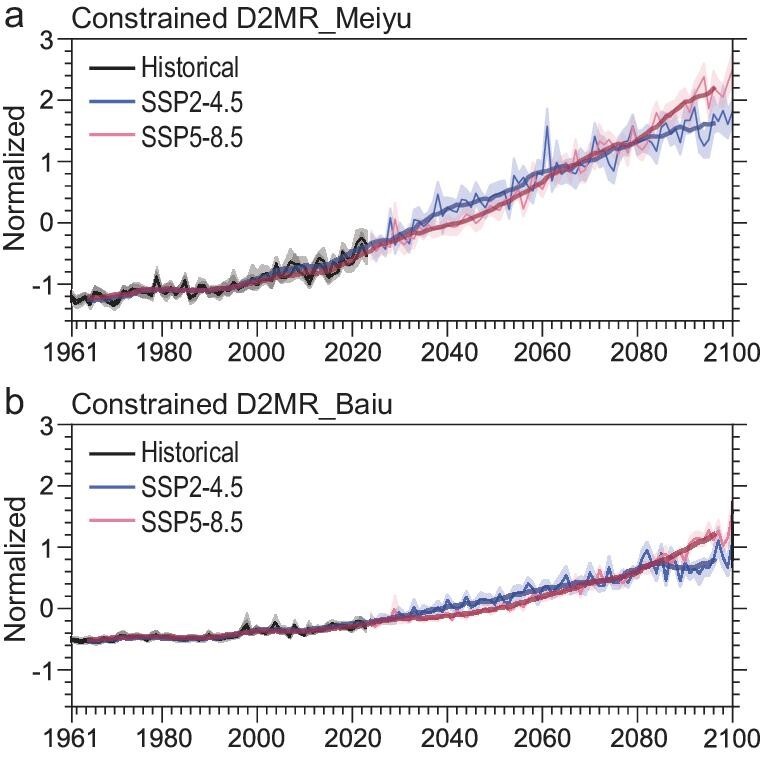
Constrained projections of D2MR in the future. Future changes in D2MR projected by using CMIP6 models in the (a) Meiyu and (b) Baiu regions under the SSP2-4.5 (blue) and SSP5-8.5 (red) scenarios. The black lines are the simulated D2MR under ALL forcings. The thick lines, solid curves and shading denote the 9-year moving average, MME and 5%–95% range of models, respectively. The box-and-whisker plots show the MME and 5%–95% likely range of projected changes by the end of this century (2081–2100) with respect to 1961–70.

In response to climate warming, the ensemble means of the D2MR projections from the CMIP6 models presented a consistently increasing trend. The changing trend of D2MR in the Meiyu area was significantly greater than that in the Baiu region, but both varied among the scenarios (Fig. 4). During 2024–2100 under the intermediate emissions scenario (SSP2-4.5), the scaled changes in D2MR exhibited a trend of 0.27/10a (*P* < 0.01) and 0.14/10a (*P* < 0.01) in the Meiyu and Baiu regions, respectively, both of which were relatively lower than their current trends. Furthermore, both regions displayed noteworthy increasing trends under the high-emissions scenario (SSP5-8.5), i.e. 0.37/10a (*P* < 0.01) and 0.20/10a (*P* < 0.01), respectively.

Before 2030, D2MR was projected to increase slightly, with a relatively smaller spread of models that are independent of scenarios. From then until ∼2060, the increase under the SSP2-4.5 scenario is shown to possibly exceed that of the SSP5-8.5 scenario in both the Meiyu and the Baiu regions, although with a certain level of noise. At this stage, both HD70AT and RI80R also showed a more obvious increasing trend under the SSP2-4.5 scenario. In contrast, CDnoR showed a decreasing trend, with a greater decline under the SSP5-8.5 scenario than under the SSP2-4.5 scenario in the Meiyu region. The rate of decline of CDnoR under the SSP5-8.5 scenario in the Baiu region was greater than that in the Meiyu region, and the trend of the change was not significant under the SSP2-4.5 scenario. Thereafter, D2MR under the SSP5-8.5 scenario increased much faster due to no mitigation of emissions, while the uptrend of D2MR tended to be much weaker under the SSP2-4.5 scenario.

By the end of the twenty-first century (2081–2100), variations in D2MR under the SSP2-4.5 and SSP5-8.5 scenarios will have produced significant differences. D2MR in the Meiyu region showed a weak increasing trend under the SSP2-4.5 scenario whereas it tended to be flat in the Baiu region. However, under the SSP5-8.5 scenario, D2MR still increased significantly. The 2081–2100 mean of D2MR was 9.3-fold more in the Meiyu region and 3.0-fold more in the Baiu region concerning the mean of 1961–70 under the SSP2-4.5 scenario. Furthermore, the influences were further enhanced to 16.7-fold and 6.4-fold, respectively, under the SSP5-8.5 scenario (Fig. 4). Despite the existence of inherent uncertainties in climate projections, the results imply a substantial surge in D2MR during a warmer future and the misty nature of the traditional Meiyu–Baiu will be close to disappearing, even in the mid-term future under moderate development and human emissions.

## DISCUSSIONS AND IMPLICATIONS

The Meiyu and the Baiu are distinct and important meteorological phenomena in East Asia and their well-known traditional features such as misty rain seem to be largely weakening along with more frequent extremes [16,29]. In this study, we constructed an index (i.e. D2MR) to quantify the changes in the traditional Meiyu–Baiu and their responses to climate change. During 1961–2023, the substantial decline of the traditional Meiyu–Baiu was clearly revealed and mainly attributable to anthropogenic warming. Moreover, under future climate-warming scenarios, both the Meiyu and the Baiu are expected to continue to stay away from their traditional characteristics. Changma in Korea possibly showed similar variations [3], although it is not fully discussed due to data dearth in this study.

Many studies have pointed out that anthropogenic influences can lead to the air holding more water at a rate of 7%/°C, cause abnormal upward motion and thus enhance rainfall intensity [30,31]. Human activities can also result in extreme dry and hot events by triggering the formation of anticyclonic regimes and the divergence of water vapor [32,33]. The differences in vertical motions and moisture conditions between stronger and weaker Meiyu–Baiu have been amplified in recent decades, indicating that dynamical and moisture conditions tend to cause extreme rainfall and drought instead of misty rainfall. More importantly, these differences also showed a significant increasing trend (Fig. S17), consistently with the D2MR changes. It is well documented that aerosol changes have played an important role in global and regional precipitation. Increasing aerosols would cause lighter rainfall, and cleaner air resulted in changes in the precipitation regime and intensity [34,35]. The local aerosol changes in East Asia were much stronger and the trend reversed from increasing to decreasing in China [36], which were supposed to largely influence regional rainfall. For example, the abrupt aerosol reductions during COVID-19 significantly contributed to the super-strong Meiyu–Baiu in 2020 [37]. However, the systematic attribution studies of local aerosol changes on the Meiyu–Baiu are still insufficient and large uncertainties need to be considered for regional projections of precipitation and its properties in future work [38].

The suspension of traditional features of the Meiyu–Baiu also bears a close relationship with strengthening climate extremes, such as rainstorms and drought, in the Yangtze River Basin and Japan islands [29,39]. Traditional Meiyu–Baiu have negative effects on human life, such as the accompanying muggy and moldy conditions, but are non-extreme and less destructive. However, it is harder for the water brought by rainstorms to be stored in soil and lakes, and this can directly cause severe damage to property and human health. Severe drought can also adversely affect land ecosystems, hydropower and so on [40,41].

Climate has a direct relationship with the historical evolution of human civilization, as different climatic conditions create different modes of production, lifestyles and cultural behavior [42]. On the scale of 1000 years, the traditional Meiyu and Baiu have shaped the civilizations and culture of China's Yangtze River Basin and Japan, but human activities have the potential to cause the traditional Meiyu–Baiu to disappear almost completely within as little as 100 years. As we know, global warming is accelerating and Earth is edging ever closer to the 1.5°C limit [43]. Over the course of history, the changing climate has led to the demise of some human civilizations, while others have taken effective countermeasures to establish new forms [44]. Anthropogenic climate changes are driving the transition from mild climate to extremes, and are also forcing humans to adapt, actively or passively [7]. How human civilization will respond to the disappearance of local traditional Meiyu–Baiu or the global enhancement of extremes is a question worthy of consideration. To address it in depth, however, requires interdisciplinary research.

This study focused more on the notable long-term changes of the traditional Meiyu and Baiu. However, D2MR also showed significant interannual-to-interdecadal variabilities. Extreme rainstorms and drought occur alternately (e.g. 2020 and 2022), presenting great challenges for climate predictions. In addition to the well-recognized western Pacific subtropical high [45], the precipitation in the Meiyu and Baiu regions are also largely affected by El Niño–Southern Oscillation [46,47], Pacific Decadal Oscillation [48] and North Atlantic Oscillation [49]. In the future, Meiyu–Baiu precipitation may be also affected by the South Asian Summer Monsoon [50]. On the basis of these sea–land–air interactions, subseasonal-to-seasonal predictions of precipitation and air temperature have also been carried out [51,52]. However, there is still no consensus on the physical mechanisms of the traditional Meiyu–Baiu on the interannual-to-interdecadal timescale. The improvement and enrichment of prediction approaches would be hugely beneficial for disaster prevention and risk management [53].

## DATA AND METHODS

### Observations and ERA5 reanalysis

Site observations of daily precipitation during 1961–2023 were employed in this study to describe the Meiyu over the Yangtze River Basin in China and the Baiu over the main islands of Japan. The data for the Meiyu region were provided by the China Meteorological Administration (https://data.cma.cn/) and those for the Baiu region were downloaded from the Global Historical Climatology Network daily (GHCNd) [54]. The stations with >15% of data missing in the Meiyu–Baiu period (from 10 June to 17 July) were removed during quality control and data for 64 stations in the Meiyu region and 18 stations for the Baiu region were ultimately included in this study. Since there are few meteorological stations in Korea containing data from 1961 to 2023 in the GHCNd data set, the Changma in Korea was not analysed in this study. Site observations of daily air temperature, wind speed and relative humidity were also used to comprehensively depict the features of the Meiyu–Baiu. Because the GHCNd data set did not include the required surface wind speed and relative humidity, the related meteorological variables in Japan were replenished from the fifth-generation reanalysis of the European Center for Medium-Range Weather Forecasts (ERA5, 1°×1°) [55]. In addition, the vertical velocity and U wind of ERA5 were used.

### CMIP6 simulations, attributions and projections

To detect and attribute the changes in the Meiyu–Baiu, the daily air temperature, precipitation, surface wind speed and relative humidity in the Coupled Model Intercomparison Project Phase 6 (CMIP6) were analysed in this study [24]. Historical (anthropogenic plus natural; ALL) and natural (NAT) simulations were analysed to extract the climate responses to different external forcings. Considering the availability of different experimental data, the following simulations were used: 65 ALL simulations from 24 models and 62 NAT simulations from 4 models (Table S3). The data for 2015–23 are supplemented by ALL and NAT forcings under the SSP 2-4.5 scenario. ANT forcing is calculated as ALL minus NAT, assuming that observational changes are independent and linear in response to various external forcings [56]. A total of 229 chunks of non-overlapping 63-year (1961–2023) simulations extracted from 24 models from the pre-industrial simulation experiment were applied to estimate the internal variability. Furthermore, we also used the model outputs under the SSP2-4.5 and SSP5-8.5 scenarios to conduct observation-constrained projections until the year 2100. For comparison and analysis, individual model simulations were preprocessed by using uniform resampling to a common horizontal grid resolution of 1° × 1° using a bilinear interpolation method. The detailed methods of the attributions and projections are available in the Supplementary Methods.

## Supplementary Material

nwae166_Supplemental_Files

## References

[1] Ding YH, Chan JCL. The East Asian summer monsoon: an overview. Meteorol Atmos Phys 2005; 89: 117–42.

[2] Tanaka M. Intraseasonal oscillation and the onset and retreat dates of the summer monsoon over east, Southeast Asia and the Western Pacific region using GMS high cloud amount data. J Meteorol Soc Jpn 1992; 70: 613–29. 10.2151/jmsj1965.70.1B_613

[3] Gao H, Yang S, Kumar A et al. Variations of the East Asian Mei-yu and simulation and prediction by the NCEP Climate Forecast System. J Climate 2011; 24: 94–108. 10.1175/2010JCLI3540.1

[4] Zhu QG, Lin JR, Shou SW et al. Principle and Methods of Synoptic Meteorology (in Chinese). Beijing: China Meteorological Press, 2003, 351.

[5] Ding YH, Liang P, Liu YJ et al. Multiscale variability of Meiyu and its prediction: a new review. JGR Atmospheres 2020; 125: e2019JD031496. 10.1029/2019JD031496

[6] Harvey W, Raymond SB. What drives societal collapse? Science 2001; 291: 609–10. 11158667 10.1126/science.1058775

[7] Li SY, Ding K, Ding AJ et al. Change of extreme snow events shaped the roof of traditional Chinese architecture in the past millennium. Sci Adv 2021; 7: eabh2601. 10.1126/sciadv.abh2601 34516886 PMC8442921

[8] Hu JF. Effects of water control on growth and development and yield of different upland rice varieties during jointing-booting stage. Crops 2020; 36: 178–82.

[9] Dunalska JA, Górniak D, Jaworska B et al. Effect of temperature on organic matter transformation in a different ambient nutrient availability. Ecol Eng 2012; 49: 27–34. 10.1016/j.ecoleng.2012.08.023

[10] Pilecky M, Meador TB, Kämmer SK et al. Response of stable isotopes (δ^2^H, δ^13^C, δ^15^N, δ^18^O) of lake water, dissolved organic matter, seston, and zooplankton to an extreme precipitation event. Sci Total Environ 2023; 891: 164622. 10.1016/j.scitotenv.2023.164622 37270009

[11] Wei WQ, Jackson GL, Adam DG et al. Regional ambient temperature is associated with human personality. Nat Hum Behav 2017; 12: 890–5. 10.1038/s41562-017-0240-0 31024181

[12] Van Tilburg WAP, Sedikides C, Wildschut T. Adverse weather evokes nostalgia. Pers Soc Psychol Bull 2018; 44: 984–95. 10.1177/0146167218756030 29537339

[13] Buckley BM, Fletcher R, Wang SYS et al. Monsoon extremes and society over the past millennium on mainland Southeast Asia. Quat Sci Rev 2014; 95: 1–19. 10.1016/j.quascirev.2014.04.022

[14] Sun B, Xue RF, Li WL et al. How does Mei-yu precipitation respond to climate change? Natl Sci Rev 2023; 10: nwad246. 10.1093/nsr/nwad246 37954193 PMC10632799

[15] Liu CH, Hu CD, Yang S et al. Extreme Mei-yu in 2020: characteristics, causes, predictability and perspectives. Earth Sci Rev 2023; 246: 104597. 10.1016/j.earscirev.2023.104597

[16] Yin ZC, Zhou BT, Duan MK et al. Climate extremes become increasingly fierce in China. The Innovation 2023; 4: 100406. 10.1016/j.xinn.2023.100406 36910135 PMC9999194

[17] Wang J, Yan R, Wu GX et al. Unprecedented decline in photosynthesis caused by summer 2022 record-breaking compound drought-heatwave over Yangtze River Basin. Sci Bull 2023; 68: 2160–3. 10.1016/j.scib.2023.08.011 37598060

[18] Miyamura K, Nawa N, Nishimura H et al. Association between heat exposure and hospitalization for diabetic ketoacidosis, hyperosmolar hyperglycemic state, and hypoglycemia in Japan. Environ Int 2022; 167: 107410. 10.1016/j.envint.2022.107410 35868079

[19] Fedorik F, Illikainen K. HAM and mould growth analysis of a wooden wall. Int J Sustain Built Environ 2013; 2: 19–26. 10.1016/j.ijsbe.2013.09.002

[20] Sun B, Wang HJ, Zhou BT et al. Interdecadal variation in the synoptic features of Mei-Yu in the Yangtze River valley region and relationship with the Pacific Decadal Oscillation. J Climate 2019; 32: 6251–70. 10.1175/JCLI-D-19-0017.1

[21] Ninomiya K, Akiyama T. Multi-scale features of Baiu, the summer monsoon over Japan and the East Asia. J Meteorol Soc Jpn 1992; 70: 467–95. 10.2151/jmsj1965.70.1B_467

[22] Duan W, Shan Z, Nikolaos C et al. Changes in temporal inequality of precipitation extremes over China due to anthropogenic forcings. npj Clim Atmos Sci 2022; 5: 33. 10.1038/s41612-022-00255-5

[23] Sun Y, Zhang XB, Ding YH et al. Understanding human influence on climate change in China. Natl Sci Rev 2022; 9: nwab113. 10.1093/nsr/nwab113 35265337 PMC8900695

[24] Eyring V, Bony S, Meehl GA et al. Overview of the Coupled Model Intercomparison Project phase 6 (CMIP6) experimental design and organization. Geosci Model Dev 2016; 9: 1937–58. 10.5194/gmd-9-1937-2016

[25] O'Neill BC, Kriegler E, Riahi K et al. A new scenario framework for climate change research: the concept of shared socioeconomic pathways. Clim Change 2014; 122: 387–400. 10.1007/s10584-013-0905-2

[26] China Meteorological Administration . Meiyu Monitoring Indices (in Chinese). Beijing: General Administration of Quality Supervision, Inspection and Quarantine of the People's Republic of China, 2017.

[27] Shih FY, Wu YT. Three-dimensional euclidean distance transformation and its application to shortest path planning. Pattern Recognit 2004; 37: 79–92. 10.1016/j.patcog.2003.08.003

[28] Xu HW, Chen HP, Wang HJ. Detectable human influence on changes in precipitation extremes across China. Earth's Future 2022; 10: e2021EF002409.

[29] Ma MM, Qu YP, Lyu J et al. The 2022 extreme drought in the Yangtze River Basin: characteristics, causes and response strategies. Geophys Res Lett 2022; 1: 162–71.

[30] Ma SM, Zhou TJ, Stone DA et al. Detectable anthropogenic shift toward heavy precipitation over eastern China. J Climate 2017; 30: 1381–96. 10.1175/JCLI-D-16-0311.1

[31] Peng DD, Zhou TJ, Zhang LX et al. Human contribution to the increasing summer precipitation in Central Asia from 1961 to 2013. J Climate 2018; 31: 8005–21. 10.1175/JCLI-D-17-0843.1

[32] Hassan WU, Nayak MA. Global teleconnections in droughts caused by oceanic and atmospheric circulation patterns. Environ Res Lett 2020; 16: 014007. 10.1088/1748-9326/abc9e2

[33] Pepler A, Dowdy A, Hope P. A global climatology of surface anticyclones, their variability, associated drivers and long-term trends. Clim Dyn 2019; 52: 5397–412. 10.1007/s00382-018-4451-5

[34] Zhou TJ, Ren LW, Zhang WX. Anthropogenic influence on extreme Meiyu rainfall in 2020 and its future risk. Sci China Earth Sci 2021; 64: 1633–44. 10.1007/s11430-020-9771-8

[35] Jiang J, Zhou TJ, Qian Y et al. Precipitation regime changes in High Mountain Asia driven by cleaner air. Nature 2023; 623: 544–9. 10.1038/s41586-023-06619-y 37821703

[36] Zhang YJ, Yin ZC, Wang HJ. Roles of climate variability on the rapid increases of early winter haze pollution in North China after 2010. Atmos Chem Phys 2020; 20: 12211–21. 10.5194/acp-20-12211-2020

[37] Yang Y, Ren L, Wu M et al. Abrupt emissions reductions during COVID-19 contributed to record summer rainfall in China. Nat Commun 2022; 13: 959. 10.1038/s41467-022-28537-9 35181650 PMC8857220

[38] Sarojini BB, Stott PA, Black E. Detection and attribution of human influence on regional precipitation. Nat Clim Change 2016; 6: 669–75. 10.1038/nclimate2976

[39] Takahashi HG, Fujinami H. Recent decadal enhancement of Meiyu–Baiu heavy rainfall over East Asia. Sci Rep 2021; 11: 13665. 34234200 10.1038/s41598-021-93006-0 PMC8263781

[40] Li XY, Piao SL, Huntingford C et al. Global variations in critical drought thresholds that impact vegetation. Natl Sci Rev 2023; 10: nwad049. 10.1093/nsr/nwad049 37064217 PMC10103823

[41] Fowler HJ, Lenderink G, Prein AF et al. Anthropogenic intensification of short-duration rainfall extremes. Nat Rev Earth Environ 2021; 2: 107–22. 10.1038/s43017-020-00128-6

[42] Tamma AC, Solomon MH. Social and economic impacts of climate. Science 2016; 353: aad9837. 27609899 10.1126/science.aad9837

[43] Matthews HD, Wynes S. Current global efforts are insufficient to limit warming to 1.5°C. Science 2022; 376: 1404–9. 10.1126/science.abo3378 35737785

[44] Degroot D, Anchukaitis K, Bauch M et al. Towards a rigorous understanding of societal responses to climate change. Nature 2021; 591: 539–50. 10.1038/s41586-021-03190-2 33762769

[45] Bao Y, Liu H, Cai X. Physical mechanism of phased variation of 2020 extremely heavy Meiyu in middle and lower reaches of Yangtze River. J Trop Meteorol 2022; 28: 273–85.

[46] Chu QC, Lian T, Chen DK et al. The role of El Niño in the extreme Mei-yu rainfall in 2020. Atmos Res 2022; 266: 105965. 10.1016/j.atmosres.2021.105965

[47] Wang SS, Yuan X, Li YH. Does a strong El Niño imply a higher predictability of extreme drought? Sci Rep 2017; 7: 40741. 10.1038/srep40741 28094328 PMC5240109

[48] Wang MY, Hu CY, Liu YH et al. Precipitation in eastern China over the past millennium varied with large-scale climate patterns. Commun Earth Environ 2022; 3: 321. 10.1038/s43247-022-00664-7

[49] Liu BQ, Yan YH, Zhu CW et al. Record-breaking Meiyu rainfall around the Yangtze River in 2020 regulated by the subseasonal phase transition of the North Atlantic Oscillation. Geophys Res Lett 2020; 47: e2020GL090342. 10.1029/2020GL090342

[50] Chen HJ, Yang S, Wei W. Future changes in the relationship between the South and East Asian summer monsoons in CMIP6 models. J Trop Meteorol 2023; 29: 191–203.

[51] Qiao SB, Chen D, Wang B et al. The longest 2020 Meiyu season over the past 60 years: subseasonal perspective and its predictions. Geophys Res Lett 2021; 48: e2021GL093596. 10.1029/2021GL093596

[52] Wang HJ, Dai YJ, Yang S et al. Predicting climate anomalies: a real challenge. Atmos Ocean Sci Lett 2022; 15: 100115. 10.1016/j.aosl.2021.100115

[53] Joshi MK, Rai A, Kulkarni A. Global-scale interdecadal variability a skillful predictor at decadal-to-multidecadal timescales for Sahelian and Indian monsoon rainfall. npj Clim Atmos Sci 2022; 5: 2.

[54] Menne MJ, Durre I, Vose RS et al. An overview of the global historical climatology network-daily database. J Atmos Ocean Technol 2012; 29: 897–910. 10.1175/JTECH-D-11-00103.1

[55] Hersbach H, Bell B, Berrisford P et al. The ERA5 global reanalysis. Q Quart J Royal Meteoro Soc 2020; 146: 1999–2049. 10.1002/qj.3803

[56] Zhang XB, Wan H, Zwiers FW et al. Attributing intensification of precipitation extremes to human influence. Geophys Res Lett 2013; 40: 5252–7. 10.1002/grl.51010

