# Supplementary material for: Traditional Meiyu–Baiu has been suspended by global warming
Source: Natl Sci Rev. 2024 May 15;11(7):nwae166. doi: 10.1093/nsr/nwae166 (PMC11173173; doi:10.1093/nsr/nwae166)
Supplement: nwae166_Supplemental_Files [file nwae166_supplemental_files.zip › Supplementary data-r.pdf]

Traditional Meiyu-Baiu has been suspended by global warming

Zhicong Yin[1,2], Xiaolei Song[1], Botao Zhou[1], Wenhao Jiang[1], Huopo Chen[2] and Huijun Wang[1,2,*]

[1]Key Laboratory of Meteorological Disaster, Ministry of Education / Collaborative Innovation Center on Forecast and Evaluation of Meteorological Disasters, Nanjing University of Information Science & Technology, Nanjing 210044, China;

[2]Nansen-Zhu International Research Centre, Institute of Atmospheric Physics, Chinese Academy of Sciences, Beijing 100029, China

***Corresponding author.** E-mail: hjwang@nuist.edu.cn

The PDF file includes:

Methods

Supplementary Tables S1–S3

Supplementary Figures S1–S17

Captions for Supplementary Movies S1

Other Supplementary Materials for this manuscript include the following:

Movies S1

METHODS

Calculation of apparent temperature

Apparent temperature (AT) is a more accurate indicator of human environmental perception [1] and integrates the meanings of thermal, humid and ventilated conditions as follows:

$$\text{AT} = 1.07T + 0.2e - 0.65V - 2.7 \quad (1)$$

$$\text{e} = \frac{RH}{100} \times 6.105 \times \exp\frac{17.27T}{237.7 + T} \quad (2)$$

where T is temperature (°C), e is vapor pressure (hPa), V is wind speed (m/s), and RH is relative humidity (%).

Detection, attribution and projection

The optimal fingerprinting technique allows us to determine whether signals can be detected in observed changes by assuming a linear model for detection and attribution analyses. The spatiotemporal variation of observations Y is calculated as the linear sum of individual external forcings X, multiplied by corresponding scaling factors (scaled fingerprint), and the internal climate variability ε estimated from the chunks from pre-industrial simulation (CTL). Detection analysis requires the reduction of both the temporal and spatial dimensions [2]. The anomalies of indexes in observations and simulations were regionally averaged to reduce the spatial dimensions. To reduce interannual variability noise, we took 3-year nonoverlapping means for all the regionally averaged time series. The simulations were masked according to the availability of observational data before the areal mean.

This method is expressed by the following equation [2,3]:

$$\text{Y} = \beta X + \varepsilon = \sum_{i=1}^{n} \beta_i x_i + \varepsilon \quad (3)$$

where Y represents the observation, β_i represents the scaling factor estimated using the total least-squares approach, x_i is the response to the ith external forcing by the multi-model

ensemble (MME) obtained by averaging each available ensemble member in each forcing, and ε denotes the regression residual representing internal variability. If the 90% confidence interval of a scaling factor is above zero, it implies that the corresponding external forcing signal can be detected in the observation. Furthermore, if the confidence interval of the scaling factor includes one, it suggests that the observed changes can be partly attributed to the external forcing. In our analysis, we determined the signal-to-noise ratios for index changes in historical simulations. The signal was derived from the linear trend in the ensemble mean for the specific index, while the noise was calculated as the standard deviation of the corresponding index in the CTL simulations [4].

Single-signal analysis involves regressing the individual model signals (ALL (anthropogenic plus natural), ANT (anthropogenic), or NAT (natural)) on the observations to assess whether the impact of forcings included in each signal can be detected in the observations.

Two-signal analysis involves regression on a linear combination of the ANT and NAT signals. This two-signal analysis method aims to assess the relative contributions of external forcings and separate the responses of different forcings.

Contributions of different signals (e.g., ANT and NAT) are converted by the ratio of these attributable and observed changes. The attributable changes are investigated by multiplying the linear trends of the model simulations by the factors and extending the results over 63 years. Furthermore, to account for uncertainty, we estimated the range (5%–95%) for the attributable change by multiplying the linear trend with the corresponding factor's marginal uncertainty range. On the other hand, the observed changes and their 90% confidence intervals (5%–95%, based on the least-squares method) were calculated as the observed trends multiplied by the corresponding 63-year temporal coverage.

Observation-constrained projection is based on the assumption that if the models underestimate (overestimate) changes in climate extremes during the historical period, similar

underestimations (overestimations) will persist in future projections [5,6]. To address this, the best estimates of scaling factors were employed to constrain overestimations in the CMIP6 projections. This was achieved by multiplying the CMIP6 ensemble projections by the scaling factors derived from the ALL responses in the single-signal diagnosis. In doing so, the method aims to improve the accuracy of future climate projections by accounting for potential biases in historical model simulations.

REFERENCES

1. Steadman RG. A universal scale of apparent temperature. *J Appl Meteorol Clim* 1984; **23:** 1674–87.
2. Ribes A, Planton S, Terray L. Application of regularised optimal fingerprinting to attribution. Part I: method, properties and idealised analysis. *Clim Dyn* 2013; **41:** 2817–36.
3. Allen MR, Stott PA. Estimating signal amplitudes in optimal fingerprinting, part I: theory. *Clim Dyn* 2003; **21:** 477–91.
4. Dong SY, Sun Y, Li C *et al.* Attribution of extreme precipitation with updated observations and CMIP6 simulations. *J Climate* 2021; **34:** 871–81.
5. Allabakash S, Lim S. Anthropogenic influence of temperature changes across East Asia using CMIP6 simulations. *Sci Rep-uk* 2022; **12:** 11896.
6. Christidis N, Stott PA, Brown SJ. The role of human activity in the recent warming of extremely warm daytime temperatures. *J Climate* 2011; **24:** 1922–30.

Table S1. Features of traditional Meiyu described by ancient poetry since the 200s AD.Key words, describing misty (blue) and moldy–muggy (red) features, are listed and translated.

Dynasty (Years)	Ancient poetry (Chinese):	Translations:
晋朝<br>Jin Dynasty<br>(265-420 AD)	夏至之雨，名为黄梅雨，沾衣服皆败黦。<br>——《阳羡风土记》	The clothes are attacked by mold and turn into yellow and black.
南北朝<br>Northern And Southern Dynasties<br>(420-589 AD)	朱帘卷丽日，翠幕蔽重阳。<br>五月炎气蒸，三时刻漏长。<br>麦随风里熟，梅逐雨中黄。<br>——庾信《奉和夏日应令》	Continuous drizzle and absent sunlight.<br>Muggy waves.
隋朝<br>Sui Dynasty<br>(581-618 AD)	黄梅雨细麦秋轻，枫叶萧萧江水平。<br>——杨广《江都夏》	Smoky drizzle.<br>Overflowed river.
唐朝<br>Tang Dynasty<br>(618-907 AD)	湛湛长江去，冥冥细雨来。<br>茅茨疏易湿，云雾密难开。<br>竟日蛟龙喜，盘涡与岸回。<br>——杜甫《梅雨》	A misty and fine rain.<br>Thatched roof is easily soaked.<br>The clouds are thick and misty rain continued for multi-days.
	海雾连南极，江云暗北津。<br>素衣今尽化，非为帝京尘。<br>——柳宗元《梅雨》	Extensively misty rain.<br>The clothes are smudged with mold.
宋朝<br>Song Dynasty<br>(960-1279 AD)	黄梅时节家家雨，青草池塘处处蛙。<br>——赵师秀《约客》	Rainy everywhere.
	漠漠轻阴拨不开，江南细雨熟黄梅。<br>——辛弃疾《鹧鸪天 败棋罚赋梅雨》	Sky is persistently overcast.<br>A fine rain comes.
元朝<br>Yuan Dynasty<br>(1271-1368 AD)	积年梅雨动兼旬，咎证源源殆有因。<br>——方回《梅雨大水》	The plum rain frequently lasts for nearly 20 days.
明朝<br>Ming Dynasty<br>(1368-1644 AD)	上梅皆当作黴，因雨当梅熟，遂讹为梅雨。<br>——《名义考》	Things turn into black during Meiyu.
	听雨原清课，虽多不厌赊。<br>——范景文《梅雨》	Continually rainy.
清朝<br>Qing Dynasty<br>(1644-1911 AD)	五月江南麦已稀，黄梅时节雨霏微。<br>——纳兰性德《浣溪沙·五月江南麦已稀》	Beautiful fine rain.
	烟霭欲明还未。但闷压、晚来天气。<br>——吴本嵩《水龙吟·梅雨》	Everything is covered in mist.<br>Weather is hot and stifling.
近现代<br>Modern China<br>(1912-1949 AD)	女贞花白草迷离，江南梅雨时。<br>阴阴帘幙万家垂。穿帘双燕飞。<br>——王国维《阮郎归·女贞花白草迷离》	Thick clouds and mist.<br>The sky is overcast.

Table S2. Features of traditional and untraditional Meiyu. Features of traditional Meiyu summarized from ancient poems (column 1) and the new features of recent untraditional Meiyu (columns 2–3). Possible quantitative meteorological variables are also included in column 4.

**Traditional Meiyu features**	**Recent untraditional Meiyu**		**Quantitative meteorological variables**
	**Heavy rain**	**Severe drought**	
Multi-days rainfall	short-time rainfall	Lack of rainfall	Consecutive rainy days
Gentle rain	Strong rainfall	Aridity	Rainfall intensity
Humid air	Abundant moisture	Dry air	Air humidity / Water vapor pressure
Muggy	Cool	Hot-dry	2m temperature / Apparent temperature
Cloudy / Less sunshine	Thick and low clouds	Sunny	Sunshine duration / solar radiation / Cloud cover

Table S3. CMIP6 simulations employed for attribution and projection. The numbers denote ensemble members (runs) for ALL, NAT, and SSP simulations and nonoverlapping chunks for CTL outputs.

CMIP6 Models	ALL/SSP245 (1961-2023)	NAT/ SSP245-NAT (1961-2023)	SSP245 (2024-2100)	SSP585 (2024-2100)	CTL
ACCESS-CM2	5		1	1	7
ACCESS-ESM1-5	4		1	1	15
CESM2-WACCM	1		1	1	7
CMCC-CM2-SR5	1		1	1	7
CMCC-ESM2	1		1	1	7
CanESM5	10	10	1	1	15
EC-Earth3	4		1	1	7
GFDL-CM4	1		1	1	7
GFDL-ESM4	1		1	1	7
HadGEM3-GC31-LL	1		1	1	7
IITM-ESM	1		1	1	3
INM-CM4-8	1		1	1	8
INM-CM5-0	1		1	1	19
IPSL-CM6A-LR	4	1	1	1	26
KACE-1-0-G	1		1	1	7
KIOST-ESM	1		1	1	2
MIROC-ES2L	1		1	1	7
MIROC6	4	50	1	1	7
MPI-ESM1-2-HR	1		1	1	7
MPI-ESM1-2-LR	10		1	1	15
MRI-ESM2-0	5		1	1	3
NorESM2-LM	3	1	1	1	7
NorESM2-MM	2		1	1	7
TaiESM1	1		1	1	7
Sum runs/chunks (models)	65 (24)	62 (4)	24 (24)	24 (24)	211 (24)

City	GDP (10 billion USD)	Population (million)
Nanjing	25.0	9.5
Shanghai	66.0	24.8
Wuhan	27.9	13.7
Hangzhou	27.8	12.2
Changsha	20.7	10.4
Hefei	17.8	9.6
Nanchang	10.7	6.5
Tokyo	94.7	14.1
Osaka	35.9	8.8
Yokohama	15.0	3.7
Nagoya	12.2	2.3
Fukuoka	6.4	1.5

Figure S1. 2022 GDP and population of several large cities in the Meiyu (green) and Baiu (blue) regions. GDP and resident population of several large cities in China (Nanjing, Shanghai, Hefei, Wuhan, Changsha, Nanchang and Hangzhou) and Japan (Fukuoka, Nagoya, Tokyo, Yokohama and Osaka) in 2022.

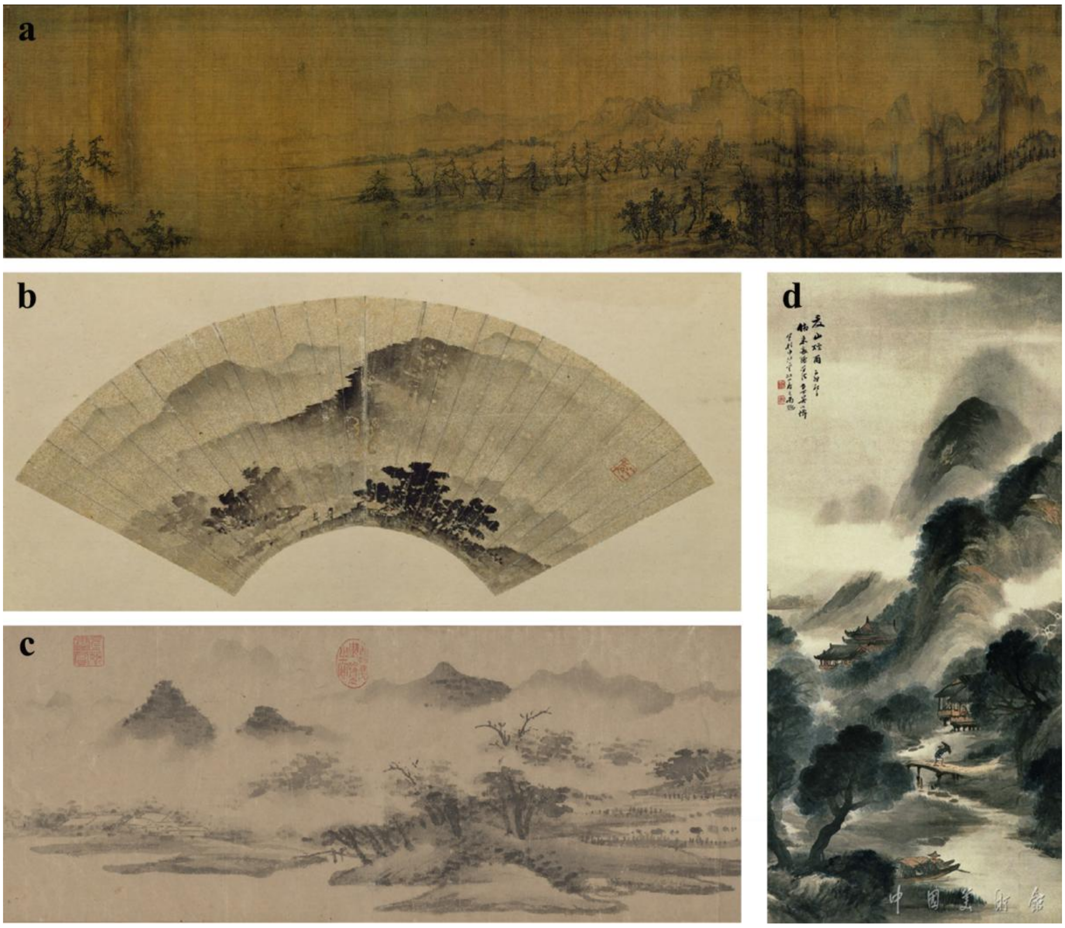

Figure S2. Examples of ancient ink and wash paintings on the theme of traditional Meiyu that are now collected by high-level national museums. (a) *Misty Rivers and Multi-Peaks* (烟江叠嶂图 in Chinese) drawn by Shen Wang in ~1084 AD of the Song Dynasty, now is collected by the Shanghai Museum. (b) *Rivers and Hills* (山水图 in Chinese) drawn by Song Jiang of the Ming Dynasty (1368−1644 AD), now is collected by The Palace Museum. (c) *Misty Rain in Suzhou Western Hill* (西山雨观图 in Chinese) drawn by Zhou Shen in ~1487 AD of the Ming Dynasty, now is collected by The Palace Museum. (d) *Misty Rain in Summer* (夏山烟雨 in Chinese) drawn by Qingyun Wu in ~1915 AD of the Qing Dynasty, now is collected by the National Art Museum of China.

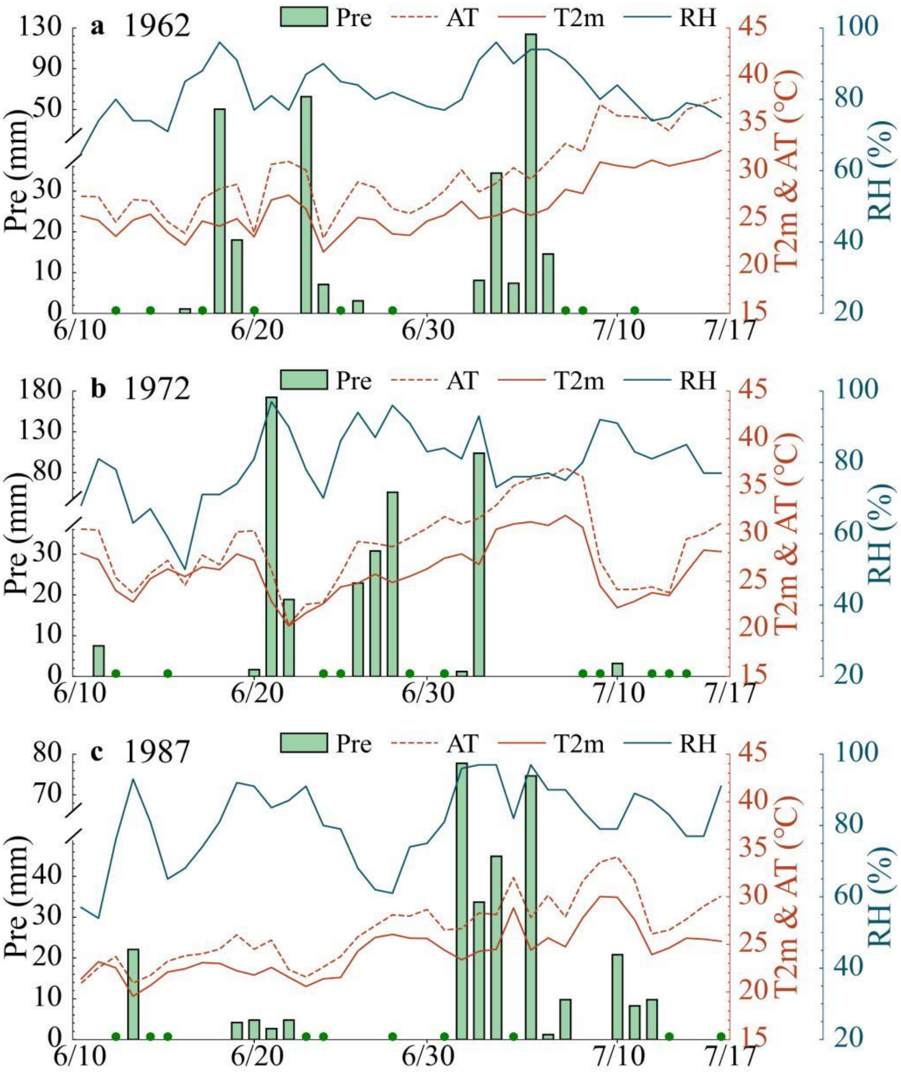

Figure S3. Examples of traditional Meiyu. Daily variations of precipitation (Pre, green column in units of mm), 2m temperature (T2m, red solid line in units of °C), apparent temperature (AT, red dashed line in units of °C), and relative humidity (RH, blue solid line in units of %) in Nanjing in 1962 (a), 1972 (b) and 1987 (c). Precipitation less than 1 mm is indicated by green dots.

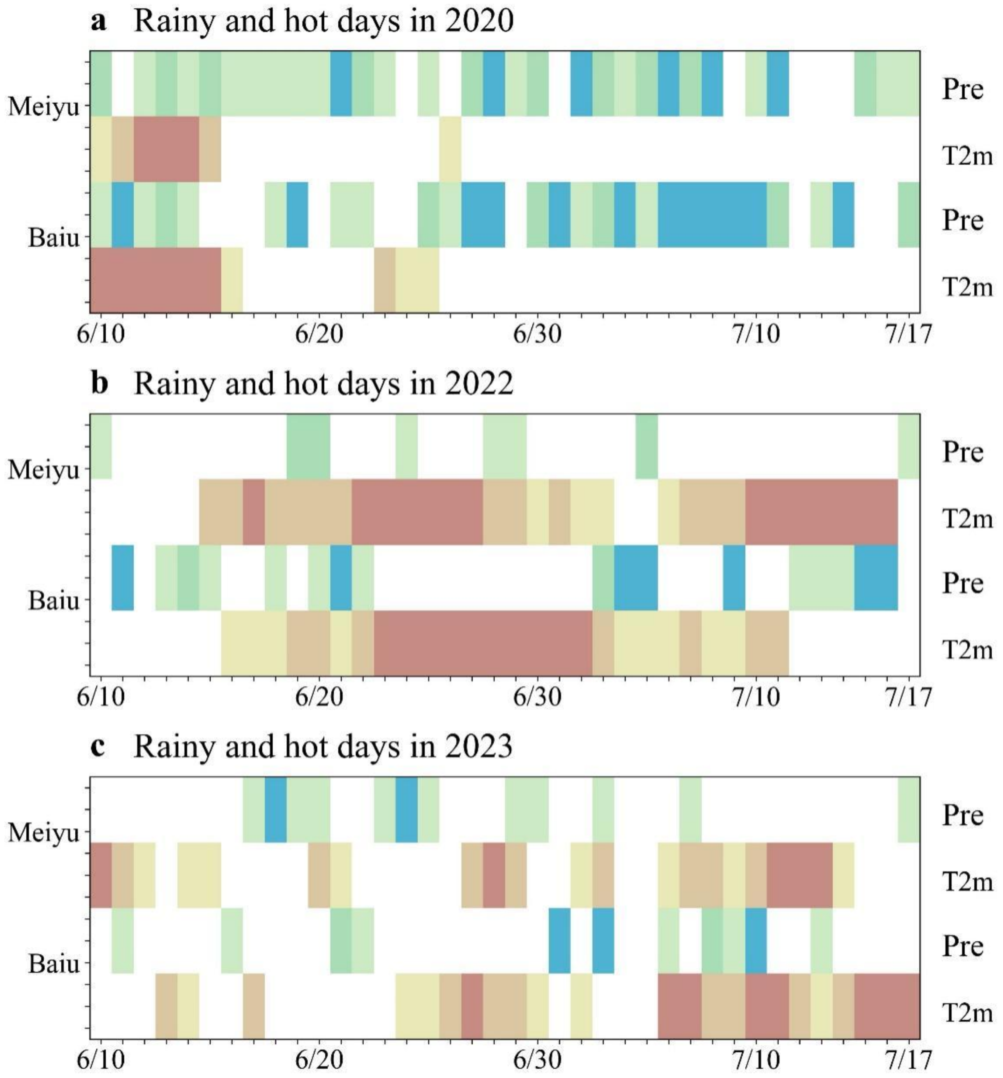

Figure S4. The Pre and T2m anomalies in the Meiyu-Baiu period. Bars colored brown from light to dark indicate that the T2m on that day in (a) 2020, (b) 2022 and (c) 2023 was greater than 75%, 85% and 95% of the climate state quantiles. Bars colored green from light to dark indicate that the Pre on that day in (a) 2020, (b) 2022 and (c) 2023 was greater than 8 mm, 15 mm and 20 mm.

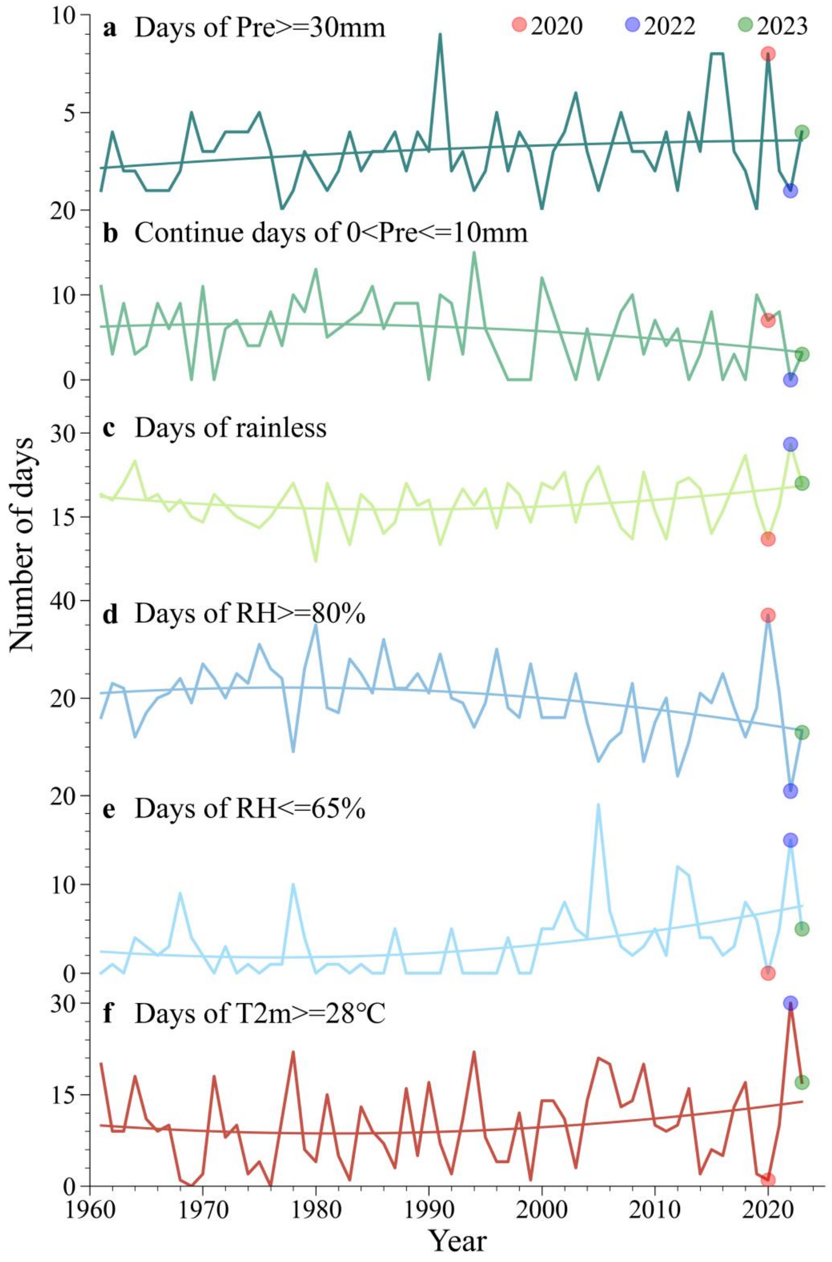

Figure S5. Variations in Pre, RH and T2m in Nanjing during the Meiyu period in 1961–2023. The number of days with Pre ≥ 30 mm (a), days with light rain lasting for 3 days or more (b), and rainless days (c); days with RH ≥ 80% (d), and days with RH ≤ 65% (e); and days with T2m ≥ 28°C (f). The quadratic fitting curves are also shown.

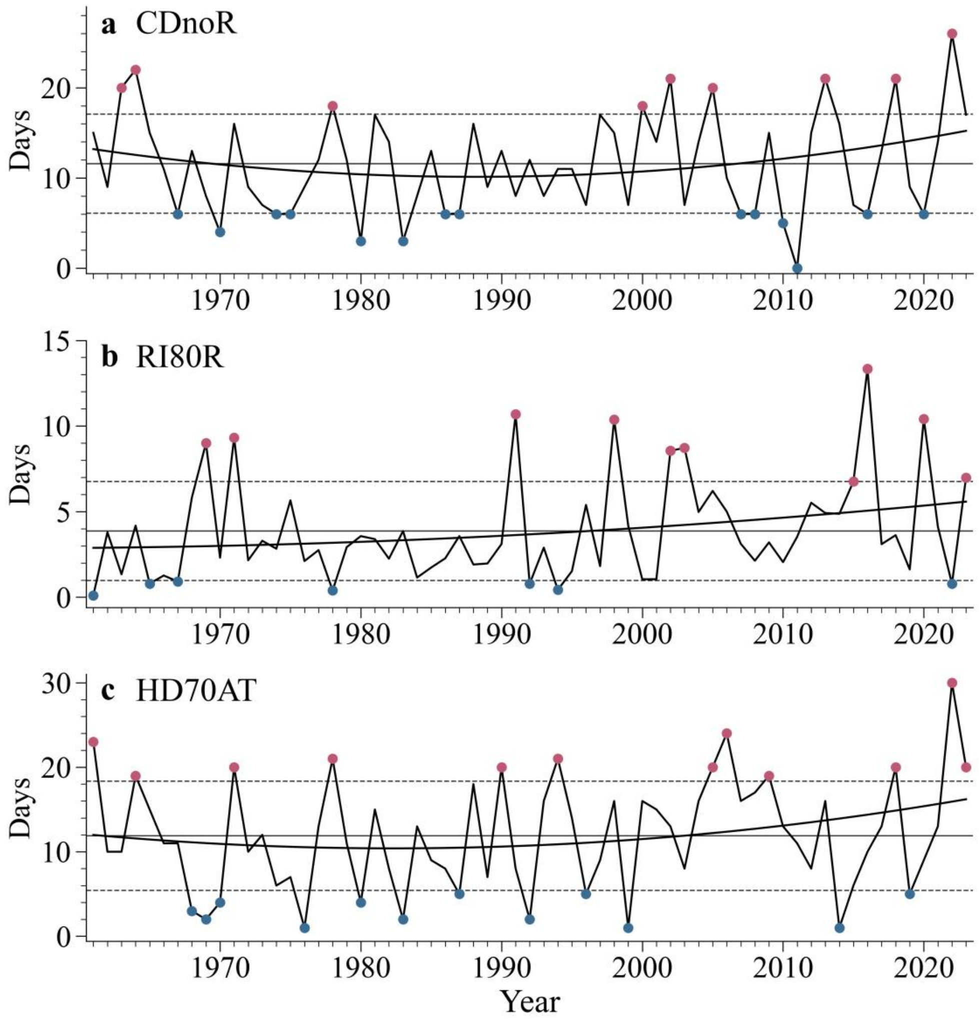

Figure S6. Variations in the three sub-indexes of D2MR and their quadratic fitting curves in Nanjing during the Meiyu period of 1961–2023. The sub-indexes include CDnoR (a), RI80R (b) and HD70AT (c). The horizontal dashed and solid gray lines represent ±1 standard deviation and the mean value, respectively. The red/green dots represent the years greater than ±1 standard deviation.

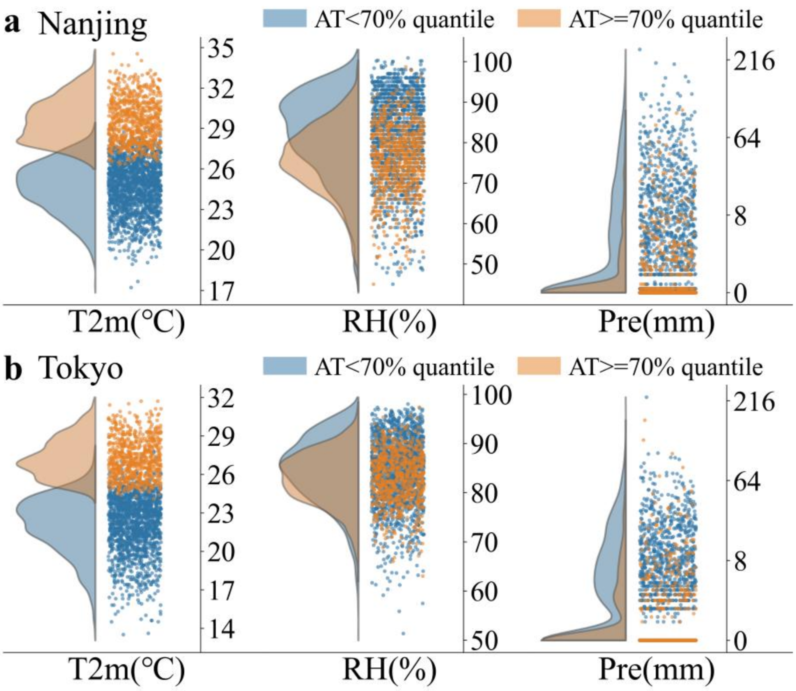

Figure S7. The differences in weather conditions when AT is greater than (orange) and less than (blue) the 70% quantile. The density distribution of T2m, RH and Pre in Nanjing (a) and Tokyo (b) were shown during Meiyu-Baiu period from 1961 to 2023.

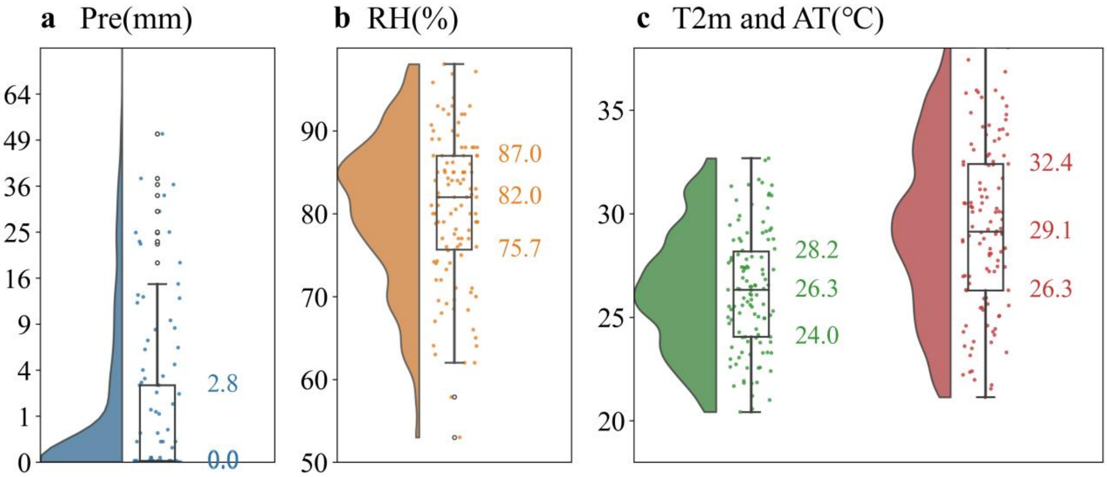

Figure S8. Features of traditional Meiyu in Nanjing. The density distribution of Pre (a), RH (b), T2m and AT (c) during the Meiyu period in Nanjing in the 3 years with the smallest D2MR index. The numbers on the right of the box plot represent 75%, 50%, and 25% quantiles, respectively.

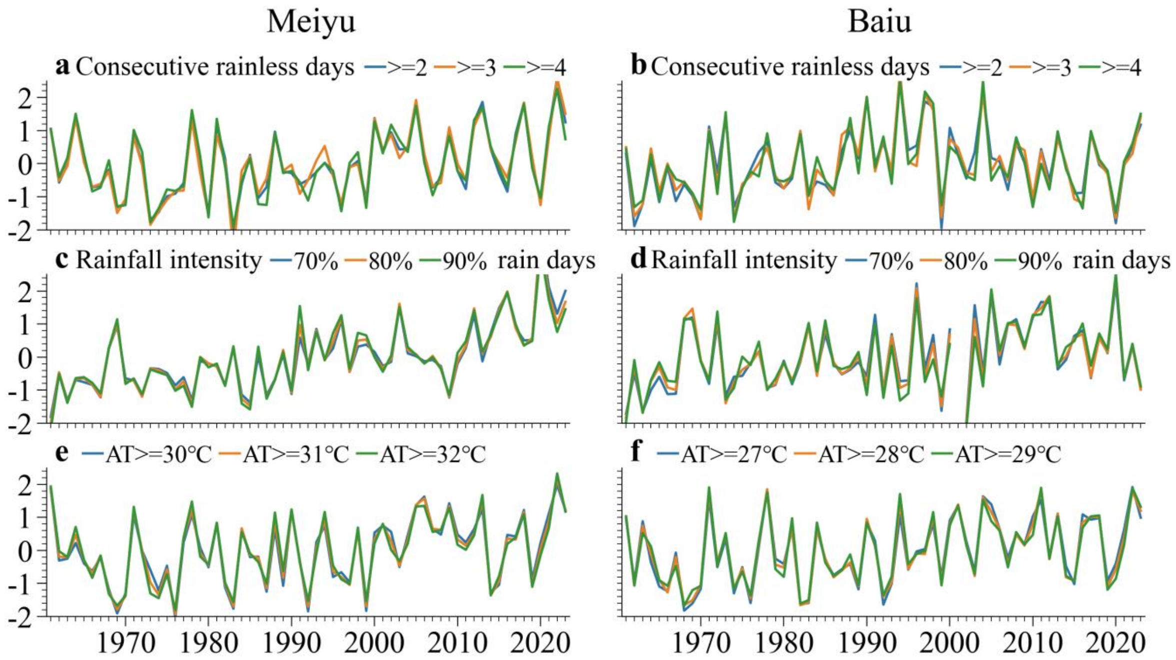

Figure S9. Variations of the three sub-indexes of D2MR with different thresholds. Sensitivity analysis included the number of consecutive days without rain exceeding 2, 3, and 4 days (a, b), rainfall intensity in 70%, 80% and 90% rain days (c, d) and the number of days with AT greater than 31, 31 and 32℃ (e, f) in Meiyu (a, c, e) and Baiu (b, d, f) region.

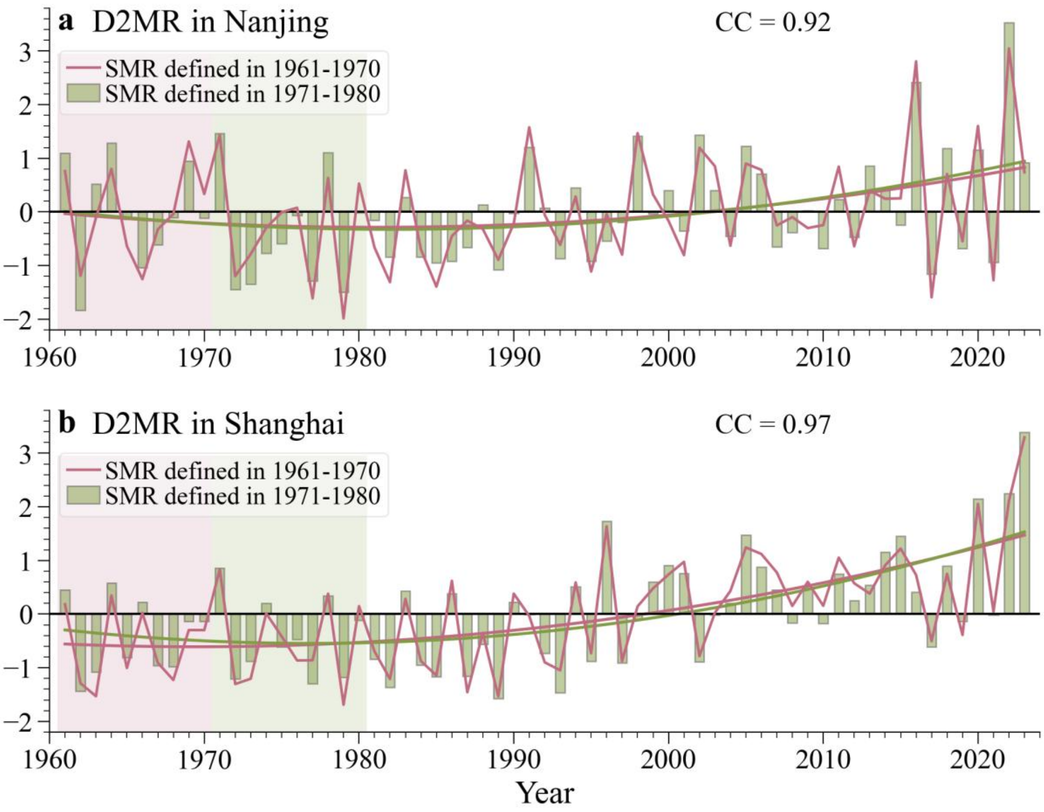

Figure S10. Variations in D2MR in Nanjing (a) and Shanghai (b) from 1961 to 2023. The SMRs are selected as 1961–1970 (solid lines) and 1971–1980 (bars), respectively, and the correlation coefficient values are also shown.

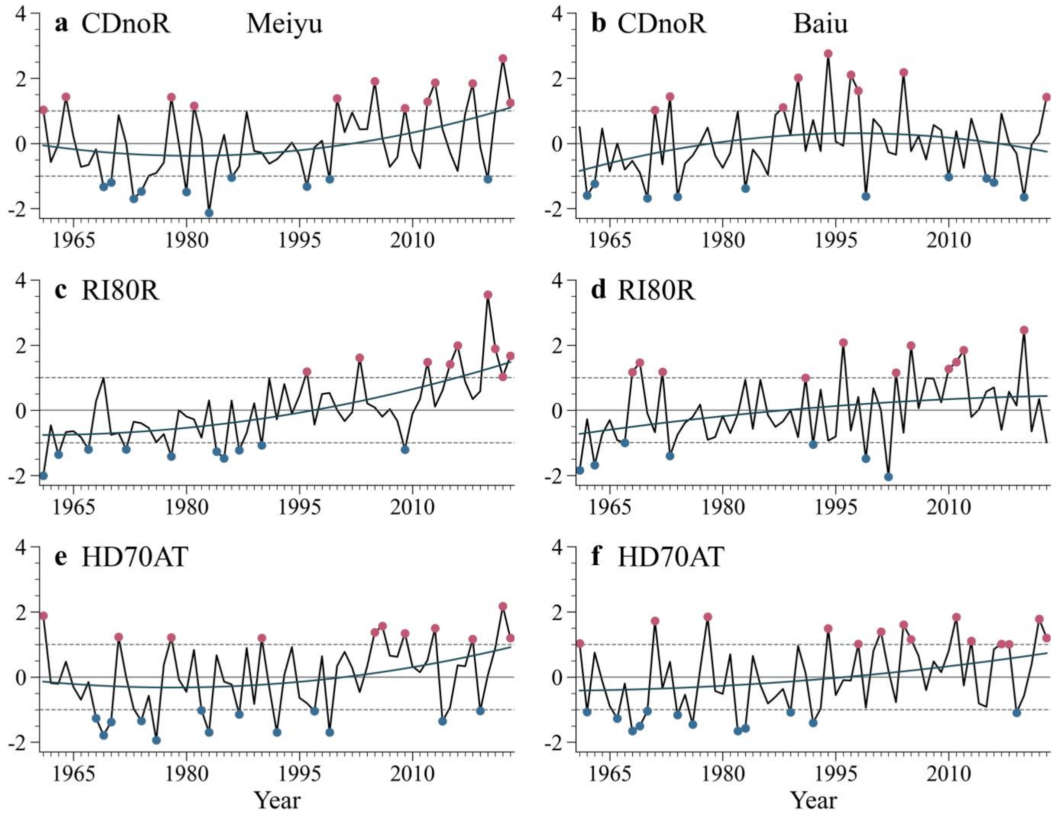

Figure S11. Variations and their quadratic fitting curves in the three sub-indexes of D2MR for the Meiyu and Baiu of 1961–2023 after standardization. The sub-indexes include CDnoR (a, b), RI80R (c, d) and HD70AT (e, f). The horizontal dashed and solid gray lines represent ±1 standard deviation and the mean value, respectively. The red/green dots represent the years greater than ±1 standard deviation.

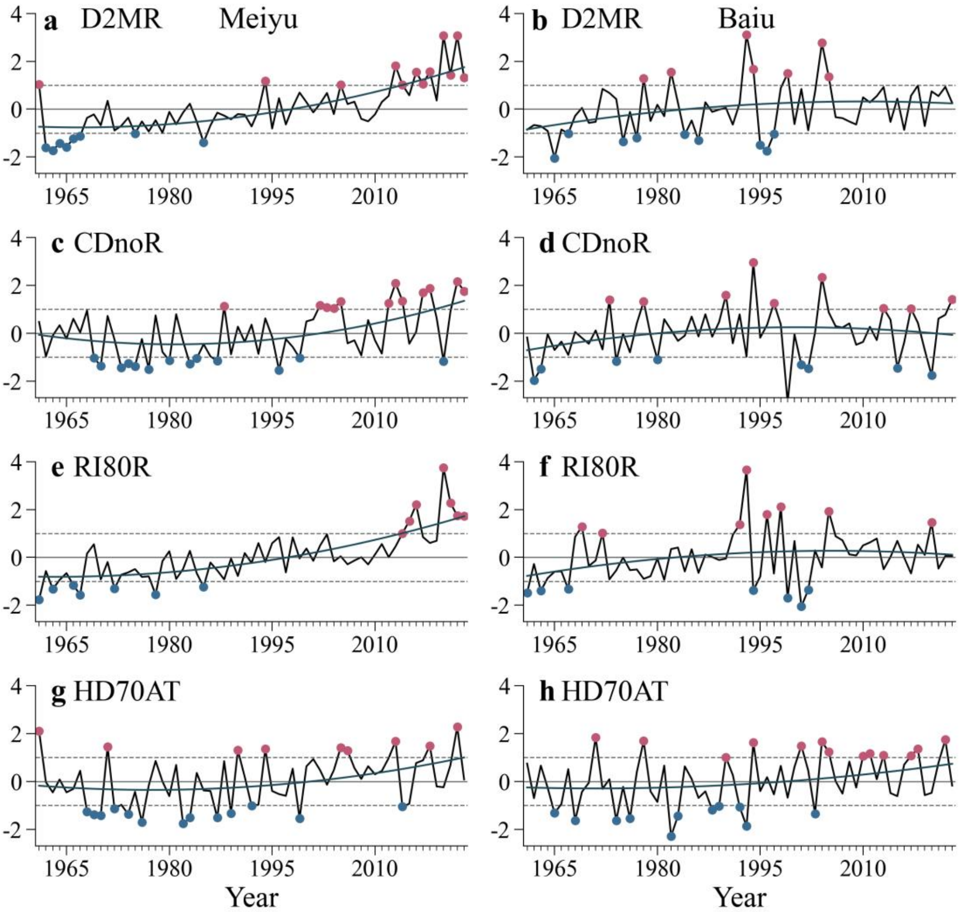

Figure S12. Variations and their quadratic fitting curves in D2MR and its sub-indexes for the Meiyu and Baiu of 1961–2023 in June and July after standardization. D2MR (a,b) and the sub-indexes include CDnoR (c, d), RI80R (e, f) and HD70AT (g, h). The horizontal dashed and solid gray lines represent ±1 standard deviation and the mean value, respectively. The red/green dots represent the years greater than ±1 standard deviation.

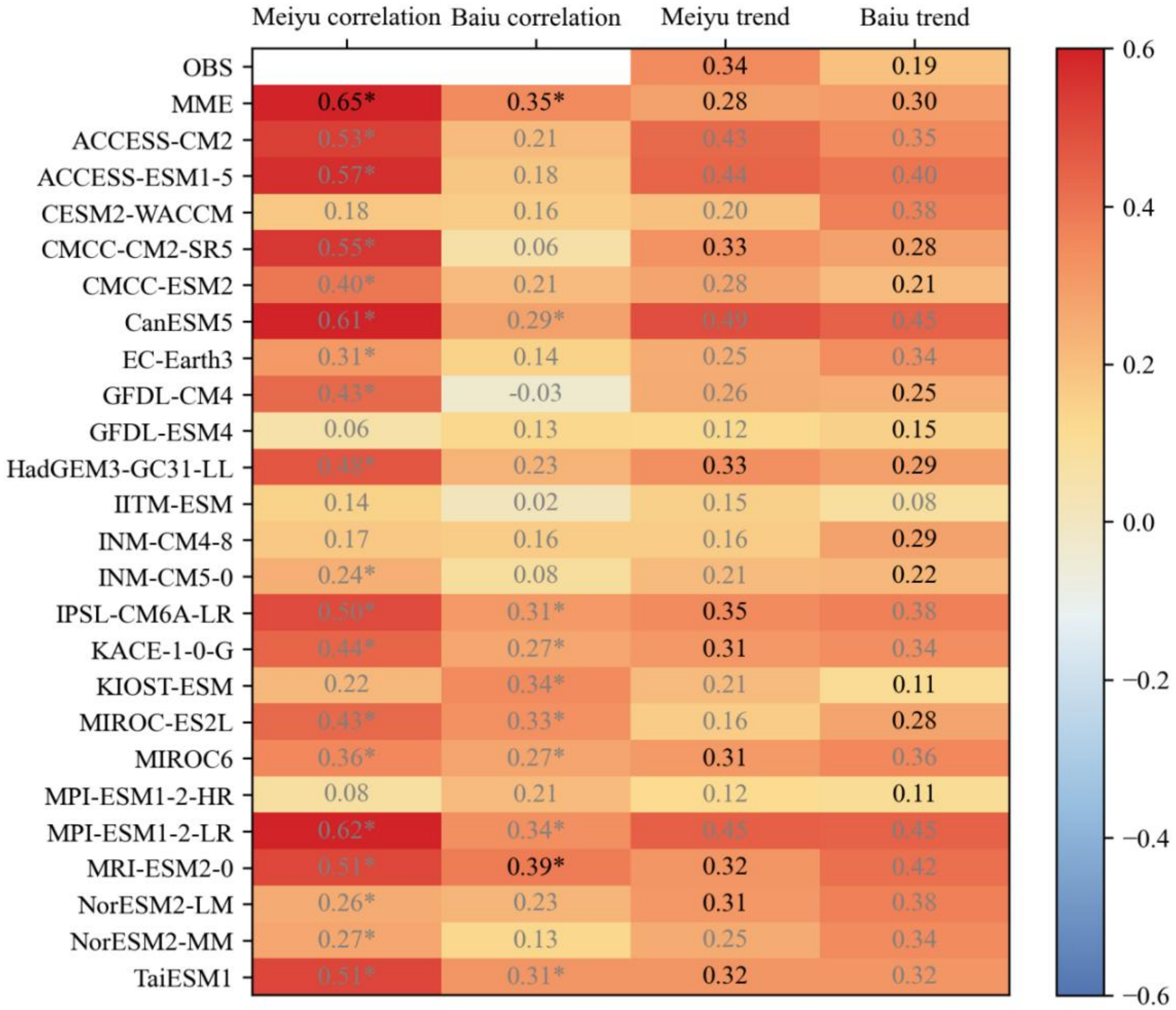

Figure S13. Performance of CMIP6 in simulating D2MR in the Meiyu and Baiu regions. The correlation coefficient of each model and their MME with observations (OBS) and the trend in D2MR during 1961–2023. Bold numbers indicate models that are closer to the OBS than MME.

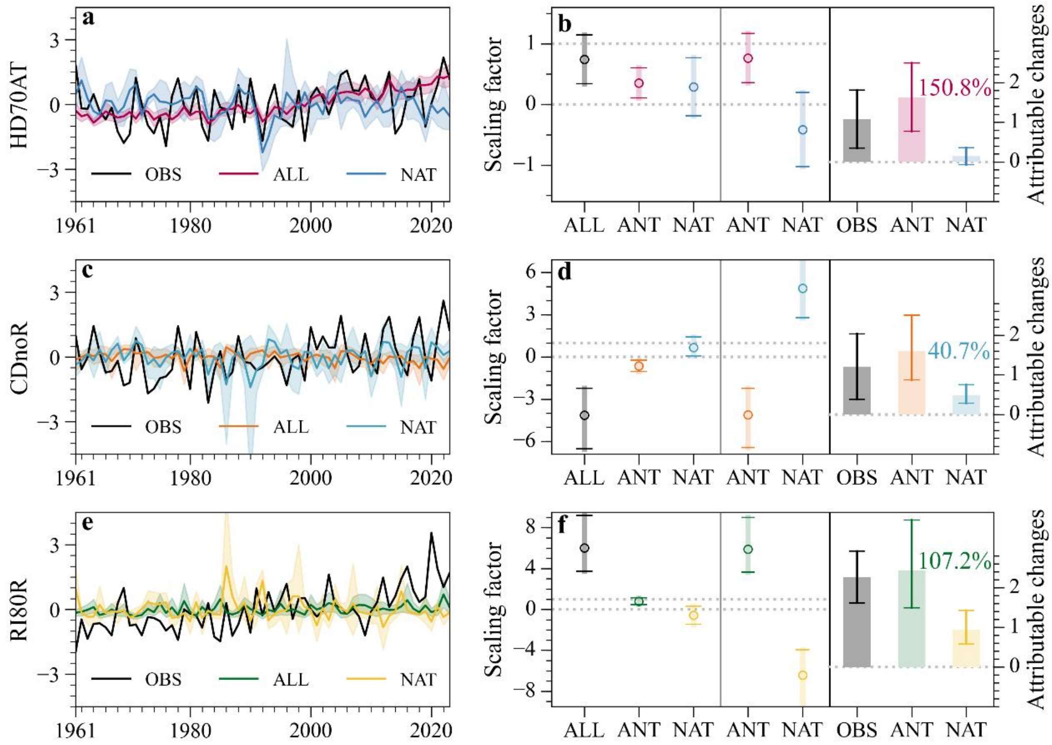

Figure S14. Attribution for the HD70AT, CDnoR and RI80R changes over the Meiyu region. HD70AT (a), CDnoR (c), and RI80R (e) in OBS and MME under the ALL and NAT forcings, and the 5% to 95% ranges of ALL and NAT ensemble simulations during 1961–2023. (b, d, f) The best estimates of the scaling factors β (data points) and corresponding 5%–95% uncertainty ranges (error bars) derived from the single-signal detection analysis (left axis) and the two-signal (ANT and NAT) analysis (middle axis). The attributable changes (right axis) originated from a two-signal analysis for the period of 1961 to 2023.

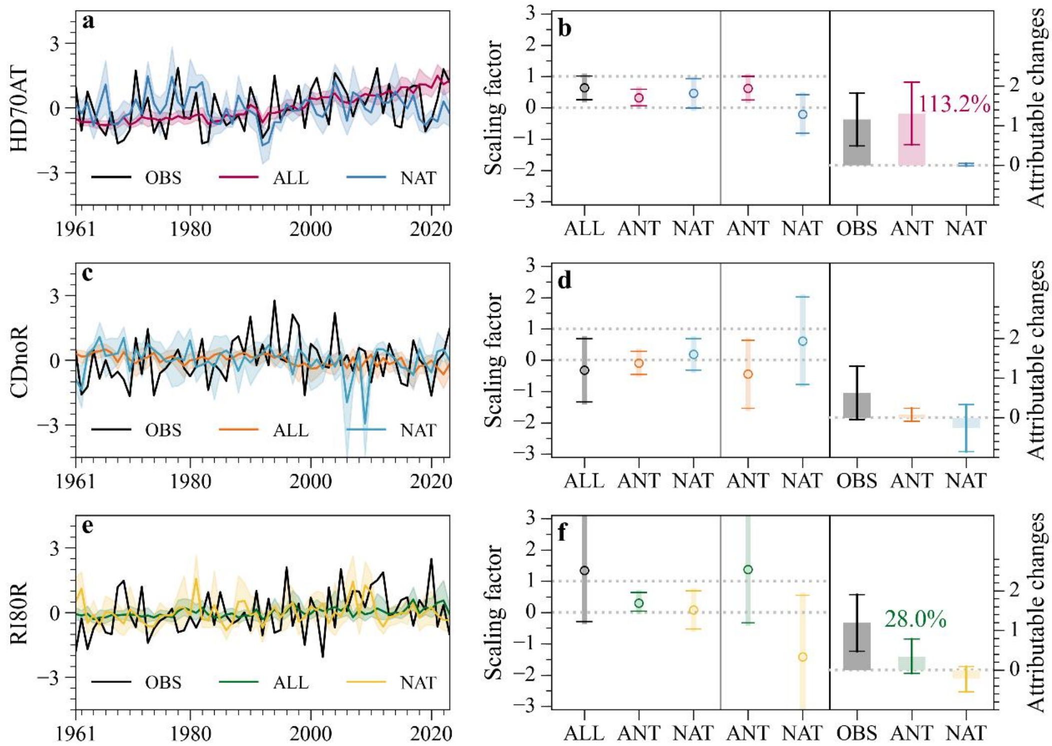

Figure S15. Attribution for the HD70AT, CDnoR and RI80R changes over the Baiu region. HD70AT (a), CDnoR (c), and RI80R (e) in OBS and MME under the ALL and NAT forcings, and the 5% to 95% ranges of ALL and NAT ensemble simulations during 1961–2023. (b, d, f) The best estimates of the scaling factors β (data points) and corresponding 5%–95% uncertainty ranges (error bars) derived from the single-signal detection analysis (left axis) and the two-signal (ANT and NAT) analysis (middle axis). The attributable changes (right axis) originated from a two-signal analysis for the period of 1961 to 2023.

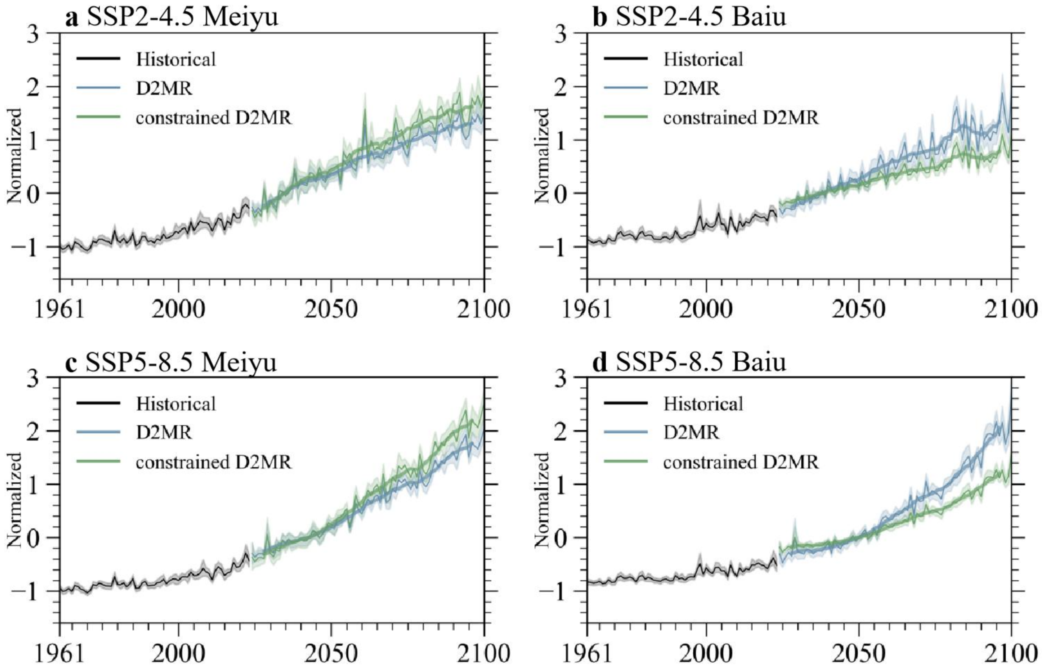

Figure S16. Multi-model mean of D2TM over the Meiyu and Baiu regions. Future changes of D2MR projected by CMIP6 models in the Meiyu (a, d) and Baiu (b, d) region under the SSP2-4.5 (a, b) and SSP5-8.5 (c, d) scenario with (green) and without (blue) observational constraints. The black lines are the simulated D2MR under ALL forcings. The thick lines, solid curves and shading denote the 9-year moving average, MME and 5%–95% range of the models, respectively.

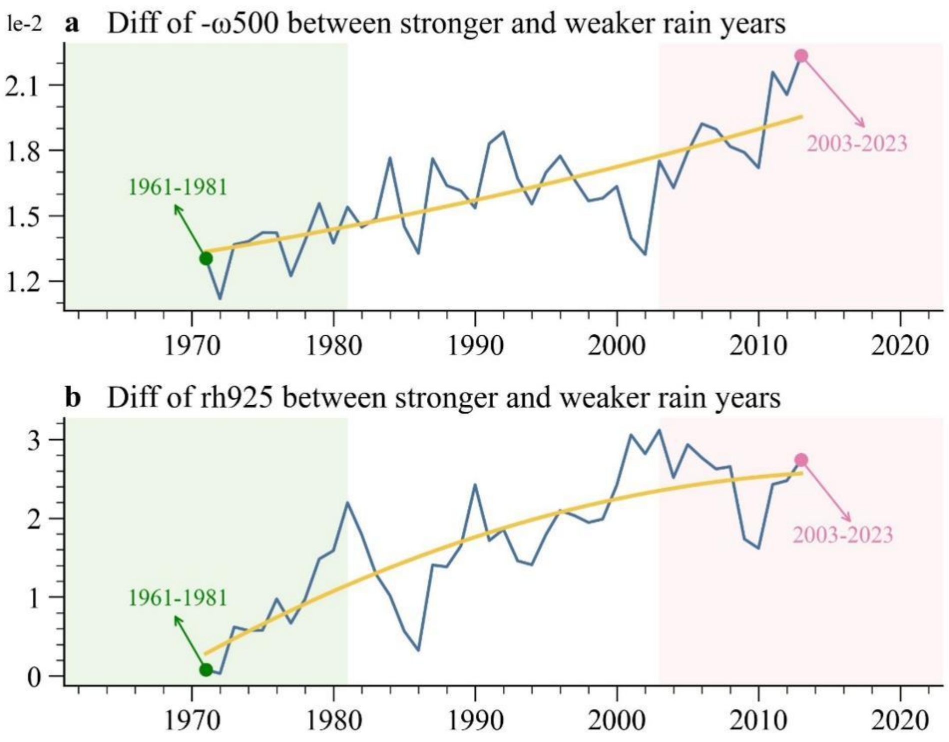

Figure S17. The differences of vertical motion and relative humidity between stronger (first 50%) and weaker (latter 50%) Meiyu-Baiu precipitation. The sliding differences of 500hPa vertical velocity (ω×-1, blue line in units of Pa/s) (a) and 925hPa relative humidity (blue line in units of %) (b) were calculated per 20 years (i.e., 10 stronger years minus 10 weaker years). Yellow lines represent the quadratic fitting curves and the upward trend passes the 99% significance test in both panel (a) and (b). Green dot represents the difference in 1961–1981, and the pink dot represents the difference in 2003–2023.

Movie S1. 3D display of D2MR in Nanjing during 1961–2023. The blue and green dots indicate years when D2MR was greater or less than ±0.2 standard deviations. The yellow rhombus indicates SMR. Red, blue and black years represent hot–dry, heavy rain and traditional Meiyu years, respectively.
